# Insect visual sensitivity to long wavelengths enhances colour contrast of insects against vegetation

**DOI:** 10.1038/s41598-021-04702-w

**Published:** 2022-01-19

**Authors:** Lu-Yi Wang, Devi Stuart-Fox, Geoff Walker, Nicholas W. Roberts, Amanda M. Franklin

**Affiliations:** 1 School of Biosciences, The University of Melbourne, Parkville, VIC 3010 Australia; 2 School of Biological Sciences, University of Bristol, Bristol, BS8 1TQ UK

**Keywords:** Ecology, Evolutionary ecology

## Abstract

The sensitivity of animal photoreceptors to different wavelengths of light strongly influence the perceived visual contrast of objects in the environment. Outside of the human visual wavelength range, ultraviolet sensitivity in many species provides important and behaviourally relevant visual contrast between objects. However, at the opposite end of the spectrum, the potential advantage of red sensitivity remains unclear. We investigated the potential benefit of long wavelength sensitivity by modelling the visual contrast of a wide range of jewel beetle colours against flowers and leaves of their host plants to hypothetical insect visual systems. We find that the presence of a long wavelength sensitive photoreceptor increases estimated colour contrast, particularly of beetles against leaves. Moreover, under our model parameters, a trichromatic visual system with ultraviolet (λ_max_ = 355 nm), short (λ_max_ = 445 nm) and long (λ_max_ = 600 nm) wavelength photoreceptors performed as well as a tetrachromatic visual system, which had an additional medium wavelength photoreceptor (λ_max_ = 530 nm). When we varied λ_max_ for the long wavelength sensitive receptor in a tetrachromatic system, contrast values between beetles, flowers and leaves were all enhanced with increasing λ_max_ from 580 nm to at least 640 nm. These results suggest a potential advantage of red sensitivity in visual discrimination of insect colours against vegetation and highlight the potential adaptive value of long wavelength sensitivity in insects.

## Introduction

Visual cues are perceived differently by different visual systems. For example, the UV sexual ornaments of male swordtails are attractive to females but are less visible to the swordtail’s main predators, Mexican tetras, because these predators lack a UV photoreceptor^1^. Variation in the detectable wavebands and perceived contrast in a visual scene is due, in part, to the number of retinal photoreceptor types and their peak sensitivities. The UV photoreceptor in birds, for instance, enhances the modelled contrast between upper and lower leaf surfaces^2^ because leaves reflect more UV light than they transmit^3^. Enhanced UV contrast could possibly facilitate leaf-localisation tasks such as prey searching. While the ecological function of UV sensitivity has been widely researched, the potential adaptive value of sensitivity to long wavelengths (600–750 nm) has received less attention (but see e.g.^4–6^).

Sensitivity to red (600–700 nm) or far red (700–750 nm) wavelengths requires the presence of a long wavelength sensitive (LWS) photoreceptor. The peak sensitivity, λ_max_, of LWS photoreceptors is generally below 600 nm, but an LWS photoreceptor often enables sensitivity to wavelengths well beyond the photoreceptor’s peak sensitivity, depending on the shape of the photoreceptor sensitivity function. For example, the human red photoreceptor peaks at 562 nm but still has 30% relative absorbance at 635 nm^7^. LWS photoreceptors that provide sensitivity beyond 600 nm are ancestral in vertebrates and common in diurnal vertebrates^8^; whereas they are usually absent in insects^9^. Nevertheless, red sensitivity has evolved independently and repeatedly in several insect families and can be found in certain species of beetles, bees, moths, and butterflies^9,10^. λ_max_ of the LWS photoreceptor is most commonly around 560–600 nm in insects^9^, with the longest known λ_max_ occurring in the butterfly, *Colias erate*, at 660 nm^11^. In the swallowtail butterfly, *Papilio aegeus*, red sensitivity enables discrimination of the young leaves preferred as oviposition sites from older, less favourable leaves^12^. Sensitivity to red or far-red could also enhance discrimination of leaves from fruit^13^, flowers, conspecific coloration or other objects in the environment which differ spectrally at long wavelengths.

Constraints on the evolution of red sensitivity depend on the mechanism used to shift photoreceptor sensitivity to longer wavelengths. Photoreceptors contain an opsin protein coupled to a light sensitive vitamin A derivative, the chromophore, which together comprise the visual pigment. Insect photoreceptors can also contain coloured filtering or screening pigments. In some insect groups such as Nymphalidae and Pieridae butterflies^11,14,15^, moths^16,17^, fireflies^18^, and click beetles^19^, a subset of photoreceptors with an LWS visual pigment have a red filter that red-shifts the receptor sensitivity to give a separate and additional red receptor. *Colias erate* butterflies, for instance, have perirhabdomal pigments that shift the peak sensitivity of the photoreceptor from around 570 nm to over 600 nm^11^. Some species use different filtering pigments with a single type of opsin to create multiple LWS photoreceptors (e.g. LWS receptors of *Pieris rapae crucivora* peak at 620 nm and 640 nm^15^). By narrowing and red-shifting the spectral sensitivity of these photoreceptors, filtering pigments inevitably reduce total light capture (i.e. absolute sensitivity). Modification to the opsin sequence can also shift peak sensitivity to longer wavelengths, but in insects this has only been documented in butterflies so far^20–22^. Changes in chromophore type, specifically use of an A2 rather than A1 chromophore, is one of the main mechanisms of increasing long wavelength sensitivity in vertebrates^23,24^, but has not been found in insects. The latter two mechanisms shift the absorption spectra of visual pigments to longer wavelengths and do not decrease absolute sensitivity. However, these mechanisms increase thermal noise because the activation energy threshold decreases with increasing λ_max_^25–30^. This increase in noise is believed to restrict the upper limit of visual pigment absorption^31^.

Here, we investigated the potential adaptive benefit of long wavelength sensitivity in insects by modelling the visual contrast of natural objects when systematically modifying visual sensitivities. To generalise the study across insect taxa, the visual sensitivities used in the models were based on the median photoreceptor peak sensitivities observed in insects. We modelled the effect of the presence or absence of an LWS photoreceptor in a trichromatic visual system and compared these to a tetrachromatic visual system. We also modelled the effect of systematically increasing the peak sensitivity of the LWS photoreceptor. We modelled increases in peak sensitivity caused by filtering pigments, as commonly found in insects, and the shifts due to modified opsin sequences for comparison. Additionally, we modelled the effect of light environment by running the same models using both daylight and civil twilight (when the sun is 0°–6° below the horizon) illumination spectra because these periods differ markedly in the spectral distribution and represent the lowest light level for colour discrimination of most diurnal insects^32^. These modelled contrasts provide an indication of how variation in visual sensitivities may influence visual discrimination, although actual discrimination for any given organism depends on colour opponent mechanisms and neural processing and can be context dependent^33^.

**Figure 1:**
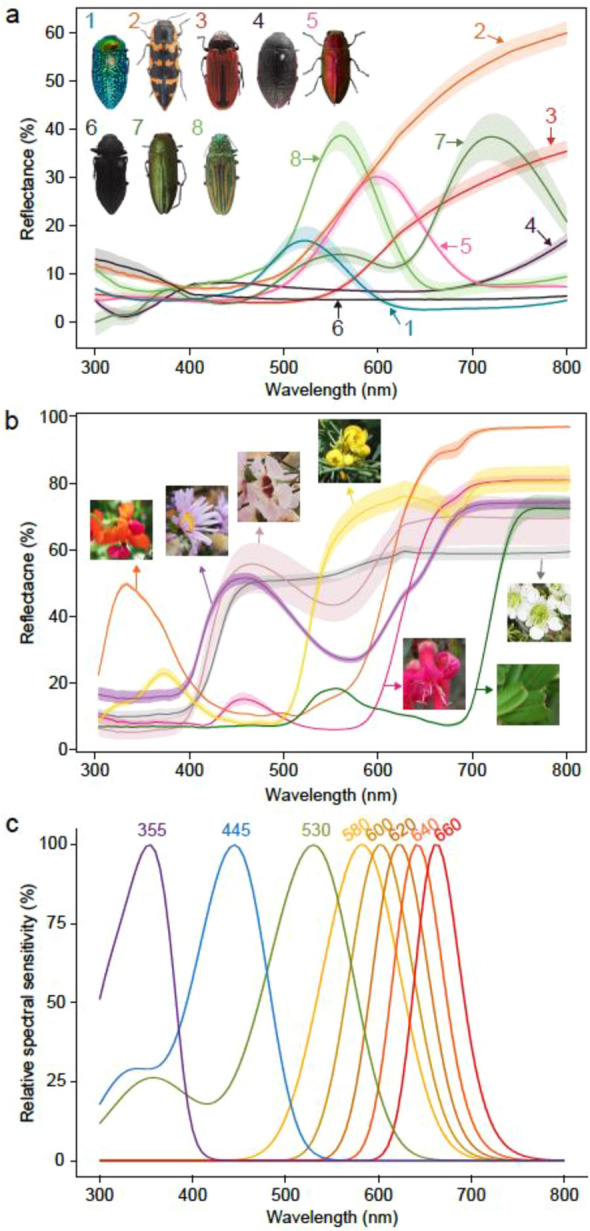
Spectral data used in visual modelling. (**a**) Reflectance spectra of representative beetle samples. Blue: *Stigmodera gratiosa*; yellow: *Cyria imperialis*; red: *Castiarina erythroptera*; violet: *Temognatha obscuripennis*; pink: *Melobasis cuprifera*; black: *Merimna atrata*; dark green: *Melobasis propinqua*; green: *Pseudotaenia gigas*. (**b**) Reflectance spectra of representative leaf and flower samples. Orange: *Chorizema cordatum*; purple: *Olearia homolepis*; pink: *Chamelaucium uncinatum*; yellow: *Senna artemisioides*; red: *Eremophila maculata*; white: *Leptospermum liversidgei*; green: *Gastrolobium bilobus*. Shaded regions in (**a**, **b)** show 95% confidence intervals of three replicates from the same species. (**c**) Sensitivity curves of photoreceptors with different peak wavelengths. (**a**–**c**) were created using R software version 3.6.3 (https://www.r-project.org) and modified using Inkscape version 1.0.2 (https://inkscape.org). Photographs by L.-Y.W.

We chose target spectra for a system where red sensitivity is likely to be ecologically relevant, namely jewel beetles (Coleoptera: Buprestidae) and their host plants. Jewel beetles are diurnally active and have diverse colours that span a broad spectral range (Fig. 1a)^34–37^. They have both pigment and structural colours that are spectrally similar to colours commonly found in other insects and are representative of the diversity of insect colours. Many species are specialists and feed on flowers or leaves of specific plant genera^38^ but the flowers of their host plants also span a diverse spectral range. Spectral data on jewel beetles and the leaves and flowers of their host plants are therefore likely to be representative of ecologically relevant visual stimuli for jewel beetles and other diurnally active insect groups. Moreover, jewel beetles have relatively large eyes and use visual signals to locate mates and host plants^39–41^. Intracellular recordings, opsin expression and electroretinogram data all indicate that jewel beetles have an LWS photoreceptor with sensitivity potentially extending into the far-red^42–44^. We first conducted a behavioural assay to confirm long wavelength sensitivity in living jewel beetles, then tested if long wavelength sensitivity enhances visual contrast using the modelling approach described above. Our spectral dataset comprises a wide range of natural spectra relevant for diurnal insects and the hypothetical visual systems represent general tri- and tetrachromatic insect vision. Hence, our results are likely to have broader relevance to tri- and tetrachromatic diurnal insects.

## Materials and methods

### Specimen collection

To obtain a broad spectral dataset of colours relevant to insects, we used jewel beetles and their host plants. Living jewel beetles were collected on bushes by both foliage beating and by hand in Victoria, Australia between November 2018 to January 2019 and October 2019. Among them, 28 individuals were collected from Wombargo (− 36.926667, 148.211111) and used in the behavioural experiment (Permit no. 10009115). We pinned the beetles soon after they deceased and measured their reflectance properties. We also used specimens of Australian jewel beetles from the Australian National Insect Collection (ANIC) to increase the colour diversity in our spectral dataset. By including both locally caught jewel beetles and samples from the ANIC collection, we could measure a broad range of jewel beetle colours from jewel beetles found across Australia. In total, we measured reflectance spectra of 34 jewel beetle species from 2 subfamilies, 8 tribes, and 13 genera with relatively even representation of different colours including human-perceived red, pink, yellow, green, blue, purple, and black (Fig. 1a; Table S2). For plant reflectance, we collected flower and leaf samples from the known host plants of Australian jewel beetles^38^ from botanic gardens in Victoria. This allowed us to obtain a diverse sample of host plants found across Australia with a broad range of flower colours. In total we sampled 46 species from 22 genera with relatively even representation across different colours, including human-perceived red, pink, yellow, purple, white, and orange. (Fig. 1b; Table S2). No blue flowers were included because we could not find any host plants with blue flowers.

### Behavioural experiments

To test the behavioural response of jewel beetles to long wavelengths, we conducted bi-choice behavioural experiments based on their positive phototaxis^45^. Full details of the experiment are presented in Supplementary Information 1. Briefly, we used a neutral-grey Y-maze as an arena with a light stimulus at the end of one arm and no stimulus at the end of the other. Beetles were tested for a response to narrow band LED lights with peak intensity at 590, 645, 680, 700, 720, and 735 nm (Marubeni, Japan; see Supplementary Information 1 for LED details). In total, 28 individuals from 2 species (16 *Castiarina dimidiata* and 12 *C. flavopicta*) completed the 6-wavelength trial series. We used the beetles’ responses (attracted/not attracted) to obtain a response curve over 590–735 nm by fitting a logistic regression for the 2 species, separately. In the model, we set the response of the beetles as the dependent variable, peak wavelength of the light source as a fixed factor, and individual ID as a random factor to account for repeated trials for each individual (see Supplementary Information 1 for results and discussion).

### Reflectance measurements and spectral processing

We collected the reflectance spectra (300–800 nm) of the beetles and host plants to model visual contrasts. Specifically, we measured the reflectance of the beetles’ elytra and major colour patches on flowers as well as the leaves of the same species using an integrating sphere (see Supplementary Information 2 for measurement details). Because of the limitation of minimum measured area, we only measured the colour patches that were larger than a 4-mm diameter circle. For each colour patch, we measured 3 different samples and averaged the 3 measurements to represent the reflectance of the colour patch. Five beetles and one flower had two colours measured because each colour patch was large enough for measurement (Fig. S4; Table S2). In total, we measured 37, 47, and 46 colour patches from beetles, flowers, and leaves, respectively.

### Hypothetical visual systems

To maximise the generality of the results across insect taxa, we created different visual systems based on the median sensitivities of insect photoreceptor types documented in a recently published review^9^. We first grouped the photoreceptors into four types based on their peak sensitivities—ultraviolet sensitive (UVS): ≤ 400 nm; short wavelength sensitive (SWS): 400–480 nm; medium wavelength sensitive (MWS): 480–560 nm; LWS: > 560 nm. Next, to avoid taxonomic bias, we calculated the median peaks of tri- and tetrachromats from each family, then calculated the median *λ*_max_ from the family medians. The representative *λ*_max_ of UVS, SWS, MWS, LWS were 355 nm, 445 nm, 530 nm, and 600 nm, respectively, and these values were used to derive spectral sensitivity functions (see below).

All spectral sensitivities were generated from peak wavelengths using an A1 chromophore template^46^, the most common chromophore in animals^47^. The longest known peak of A1-based visual pigments in animals is ca. 570 nm^48–51^. In most insects with peak sensitivities beyond 570 nm, the shift to longer sensitivity is achieved through red or orange pigments which act as filters^11,44,52,53^ (but see *Papilio* and *Eumaeus* that shift through opsin sequence modification^21,22^). Therefore, we generated LWS spectral sensitivity curves by applying cut-off filters to the 570 nm A1-based profile (Fig. S5; Supplementary Information 3). The cut-off filter was selected to achieve a similar spectral profile as the LWS photoreceptor of other insects^11,44,52,53^. Additionally, we generated another set of LWS spectral sensitivities with the same *λ*_max_ values directly from the template. This equates to shifts in *λ*_max_ due to changes in the sequence of the opsin protein and results in much broader LWS sensitivity curves (Supplementary Information 3). This enabled us to compare the effect of sensitivity shifts generated from different mechanisms on visual contrast, though the opsin shifts that we have modelled (λ_max_ = 580 nm, 600 nm, 620 nm, 640 nm, 660 nm) are longer peak sensitivities than recorded for any insect opsins.

To test whether the presence of an LWS photoreceptor enhances visual contrast, we created three trichromatic visual systems that varied in their photoreceptor composition, based on common insect photoreceptor combinations [Fig. 1c; USM (UVS, SWS, MWS); UML (UVS, MWS, LWS); USL (UVS, SWS, LWS)] we identified from van der Kooi et al.^9^. We did not create a visual system without the UV photoreceptor because few, if any, tri- or tetrachromatic insects do not have a UV receptor^9^. Additionally, we modelled a tetrachromatic visual system USML (UVS, SWS, MWS, LWS) to compare with the three trichromatic visual systems because discrimination has been reported to increase by tetrachromacy, especially an additional LWS photoreceptor^54^.

Next, we tested if increasing the peak sensitivity of the LWS photoreceptor increased discriminability between targets. We created five tetrachromatic visual systems (VS), where we systematically filter-shifted the LWS photoreceptor 20 nm from 580 nm to the longest known peak (660 nm in a butterfly)—VS 580, VS 600, VS 620, VS 640, VS 660 (Fig. 1c). Shifting LWS peak sensitivity while keeping other photoreceptors constant changes the overlap of sensitivity curves. To evaluate the effect of overlap in sensitivities on results, we ran the model using an evenly spaced visual system (λ_max_ of receptors: 355 nm, 455 nm, 560 nm, 660 nm) and compared the contrasts with that of VS 660 (Supplementary Information 4).

### Visual modelling

For each of the above visual systems, we estimated visual contrast of pairs of reflectance spectra (comparison groups: *‘flower vs. leaf’*, *‘beetle vs. leaf’*, *‘beetle vs. flower’*) using the receptor-noise limited model^55,56^ implemented in the R package ‘pavo’^57^. This model assumes that colour discrimination is limited only by photoreceptor noise and ignores effects of higher level processing. Colour contrasts are expressed in units of just-noticeable-differences (JND), where 1 JND is the theoretical threshold of colour discrimination for stimuli viewed simultaneously under ideal viewing conditions^56^ (see “Discussion” section regarding factors affecting actual discrimination thresholds).

We first calculated the quantum catch of each photoreceptor under standard daylight illumination (D65; Commission internationale de l’éclairage—CIE) using the ‘vismodel’ function with von Kries chromatic adaptation and average leaf reflectance as the adapting background. Next, we calculated the chromatic contrast between pairs of spectra using the function ‘coldist’ (with signal in each photoreceptor proportional to the natural logarithm of the quantum catch, in accordance with Fechner’s law). For the tetrachromat, we used a Weber fraction of 0.12^56^ to account for the receptor noise of the LWS photoreceptor. We used relative photoreceptor density of 1.14, 1, 1.26, and 1.38 for the four photoreceptors (UVS, SWS, MWS, and LWS, respectively) based on relative opsin gene expression reported in a jewel beetle, the Emerald ash borer (*Agrilus planipennis*)^43^. For the trichromatic visual systems, we ensured the same number of photoreceptors within a neurally integrative unit as the tetrachromatic visual system. Specifically, we proportionally redistributed photoreceptors from the missing receptor class between the remaining three classes while keeping the noise level of individual photoreceptors the same across visual systems, thereby improving their signal-to-noise ratios^54^ (Supplementary Information 5).

To test whether our results were sensitive to the photoreceptor ratios used, we repeated analyses using published ratios that represent the known variation in tetrachromatic insects, which happen to be two butterflies (*Papilio xuthus*—1.00: 1.00: 4.08: 2.92^58^; *Heliconius* sp. type III—1.00: 1.44: 2.22: 11.11^59^; details in Supplementary Information 6). To test if the contrast values change in different light environments, we ran the same vision models but using civil twilight for illumination (Supplementary Information 7). We chose twilight illumination because it has the greatest difference in spectral distribution from daylight and has proportionally more blue and red light compared with daylight illumination in the 300–800 nm range (300–500 nm, daylight: 37%, twilight: 42%; 650–800 nm, daylight: 38%, twilight: 41%). Moreover, it is likely close to the dimmest light level for colour discrimination of diurnal insects^32^. We took photon shot noise into account when using twilight illumination and converted the absolute irradiance of twilight into photon flux for twilight models.

### Statistical analysis

To test how different photoreceptor combinations influence visual contrast between beetles, flowers and leaves, we compared chromatic contrast values among the four visual systems—USM, UML, USL and USML for each comparison group (*‘flower vs. leaf’*, *‘beetle vs. leaf’*, *‘beetle vs. flower’*). For each comparison group, we ran a generalised linear mixed model (GLMM; ‘lmer’ function in the R package ‘lme4’^60^) with JNDs for each pairwise combination of spectra as the dependent variable and visual system as the fixed factor. Since each spectrum was compared to multiple others (e.g. each beetle spectrum to each of the flower spectra in the *‘beetle vs. flower’* comparison group) we included two random effects in each model, one for each type of spectra being compared (e.g. beetle ID and flower ID). Subsequent post hoc Tukey honest significant differences (Tukey HSD) with Bonferroni correction were applied to test for significant differences between each pair of visual systems. We used the same approach to test the effect of visual systems with an increase in λ_max_ from 580 to 680 nm (20 nm increments). All statistical analyses were performed in R 3.6.3^61^.

## Results

### Visual sensitivity at long wavelengths in living beetles

Behavioural experiments confirmed red sensitivity in both species of jewel beetle and possibly far-red sensitivity in *C. dimidiata*. 96% of jewel beetles were attracted by light at 590 nm and ca. 87% of them still responded to light at 645 nm (*C. dimidiata*: 100% and 96%; *C. flavopicta*, 93% and 78%; at 590 nm and 645 nm) (Fig. S1). The 50% response rate was at ca. 704 nm for *C. dimidiata* and ca. 685 nm for *C. flavopicta*. There was only one *C. dimidiata* individual that responded to light at 735 nm, which might be random movement. Overall, compared to the dark arm, *C. dimidiata* and *C. flavopicta* beetles reached the ’choice zone’ of the lit arm 19 and 5 times more often, respectively. This indicated that the valid choices of the beetles were not random movement.

### Varying photoreceptor combinations

**Figure 2:**
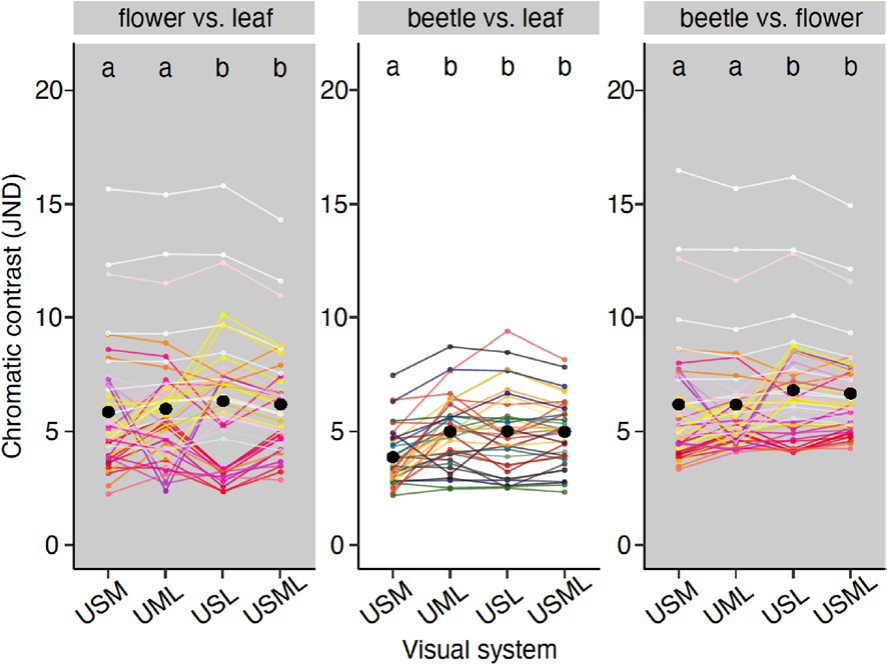
Comparison of chromatic contrast for visual systems with different photoreceptor combinations: USM (UVS, SWS, MWS), UML (UVS, MWS, LWS), USL (UVS, SWS, LWS), USML (UVS, SWS, MWS, LWS). Black dots show the means of the representative contrast values in each visual system. Each coloured dot represents the average contrast value of each flower pattern to all leaves (left panel), each beetle colour to all leaves (middle panel) or each flower colour to all beetle colours (right panel). Colours of the dots correspond to the human-visible colour of the flower (left and right panels) or beetle (middle panel) with the lines of the same colour connecting the results between different visual systems. This is for graphical representation only; statistical tests are based on all pairwise combinations of spectra and not averages. Letters on the top of each panel show the significant difference in contrast value between visual systems. Three contrast values > 10 JND are from flowers (2 white and 1 light pink) that have high UV—blue chroma compared to beetles and leaves. The Figure was created using R software version 3.6.3 (https://www.r-project.org) and modified using Inkscape version 1.0.2 (https://inkscape.org).

We found contrast values were significantly different between visual systems for all comparison groups (Fig. 2, Table 1). The presence of both short and long wavelength sensitive receptors together improved the contrast of beetles against flowers and flowers against leaves. The USL and USML visual systems, which have both short and long wavelength sensitive receptors, had the same and significantly higher contrasts than the other two visual systems (USM and UML) for these two comparison groups (Tukey HSD, all *p* < 0.001; except UML-USML in ‘*flower vs. leaf*’, *p* < 0.01). USL and USML increased contrast values by 7% on average. There was no difference between USM and UML in comparisons of beetles against flowers and flowers against leaves (*p* = 0.09 and 1.00, respectively).

**Table 1:** Wald Chi-square test results among contrasts of visual systems in different comparison groups.

Comparison group	Varied photoreceptor combination	Shifts in the peak of long wavelength sensitivity
Flower versus Leaf	χ^2^ = 78.74, df = 3, *p* < 0.001	χ^2^ = 2267.57, df = 4, *p* < 0.001
Beetle versus Leaf	χ^2^ = 665.32, df = 3, *p* < 0.001	χ^2^ = 2119.30, df = 4, *p* < 0.001
Beetle versus Flower	χ^2^ = 114.99, df = 3, *p* < 0.001	χ^2^ = 607.88, df = 4, *p* < 0.001

The presence of a long wavelength sensitive receptor improved the contrast of beetles against leaves. In this comparison (‘*beetle vs. leaf*’), there was no significant difference between UML, USL, and USML (all *p* = 1.00; Fig. 2) and USM had a significantly lower contrast value than the other three visual systems by around 29% (all *p* < 0.001). Additionally, we found no advantage to tetrachromacy over trichromacy where USL had the same contrast values as USML for all comparison groups (‘*flower vs. leaf*’, *p* = 0.12; ‘*beetle vs. leaf*’, *p* = 1.00; ‘*beetle vs. flower*’, *p* = 0.28; Fig. 2).

Models where changes in the spectral absorbance of the long wavelength sensitive receptor were due to modified opsin sequences rather than the optical influence of screening pigments showed qualitatively similar results for the comparison of beetles against leaves, with a long wavelength sensitive receptor improving contrast values (Fig. S12). However, only the presence of a short wavelength sensitive receptor (rather than both short and long wavelength sensitive receptors together) improved contrast values of beetles against flowers and flowers against leaves (full results in Supplementary Information 8).

The contrast values obtained from the four visual systems in the models using civil twilight showed different patterns and were substantially lower than the values obtained from the models using daylight (Fig. S9). In twilight illumination, USM performed significantly worse than the other visual systems and the tetrachromat performed significantly better than the trichromats but all contrast values were below 2 JND (Supplementary Information 7).

There were two white and one pink flower (*Eremophila laanii*, *Actinotus helianthi*, and *Chamelaucium uncinatum*) with exceptionally high contrast values when compared against beetles and leaves (Fig. 2). Such high contrast was the result of a steep rise in reflectance at ca. 400 nm in these three flowers, which was not present in beetles and leaves.

### Shifts in the peak of long wavelength sensitivity

**Figure 3:**
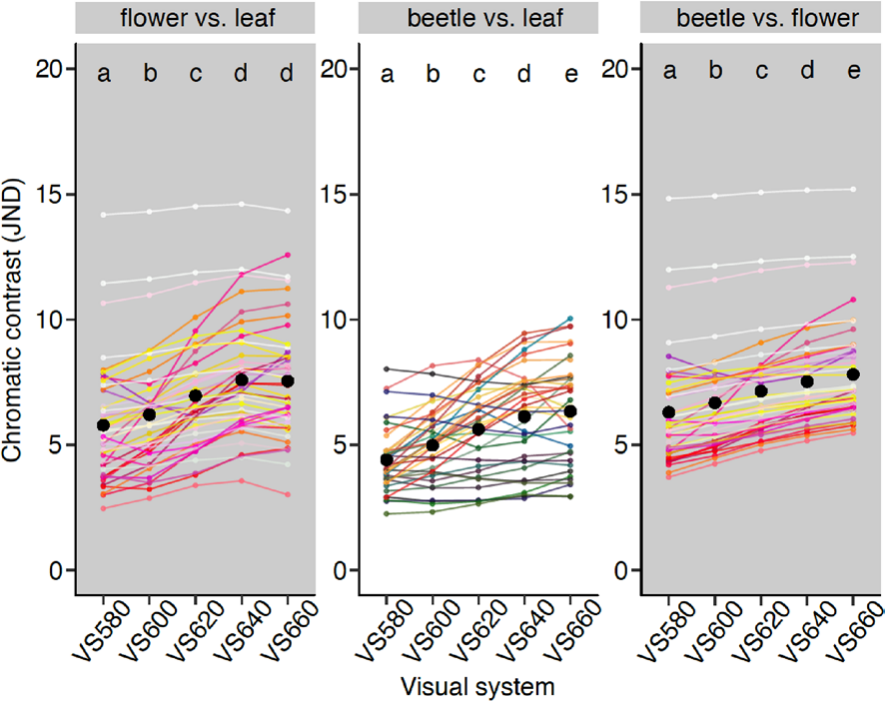
Comparison of chromatic contrast between visual systems (VS) with the long wavelength sensitive photoreceptor peaking at different wavelengths. Black dots show the means of the representative contrast values in each visual system. Each coloured dot represents the average contrast value of each flower pattern to all leaves (left panel), each beetle colour to all leaves (middle panel) or each flower colour to all beetle colours (right panel). Colours of the dots correspond to the human-visible colour of the flower (left and right panels) or beetle (middle panel) with the lines of the same colour connecting the results between different visual systems. This is for graphical representation only; statistical tests are based on all pairwise combinations of spectra and not averages. Letters on the top of each panel show the significant difference in contrast value between visual systems. Three contrast values > 10 JND are from flowers (2 white and 1 light pink) that have high UV—blue chroma compared to beetles and leaves. The Figure was created using R software version 3.6.3 (https://www.r-project.org) and modified using Inkscape version 1.0.2 (https://inkscape.org).

Contrast values increased as the peak sensitivity of the long wavelength sensitive photoreceptor increased from 580 nm to at least 640 nm for all comparison groups (Fig. 3; Table 1). In the *‘beetle vs. flower’* comparison, the contrast value steadily increased from VS 580 to VS 660 (stepwise increase in average contrast value by 6%, 7%, 5%, 4% as the long wavelength sensitive peak increased from 580 to 660 nm; *p* < 0.001 for all pairwise comparisons). Similarly, in the *‘beetle vs. leaf’* comparison, the contrast value increased from VS 580 to VS 660 but more steeply (stepwise increase in average contrast value by 13%, 13%, 9%, 3% as the long wavelength sensitive peak increased from 580 to 660 nm; *p* < 0.001 for all pairwise comparisons). The average contrast value of VS 640 was 39% higher than that of VS 580. For the *‘flower vs. leaf’* comparison, contrast values increased steeply from VS 580 to VS 640 (stepwise increase in average contrast value by 7%, 12%, 9%, as the long wavelength sensitive peak increased from 580 to 640 nm; *p* < 0.001 for all pairwise comparisons) but there was no significant difference between VS 640 and VS 660 (*p* = 1.00). Results were qualitatively the same for different photoreceptor ratios (Supplementary Information 6), indicating that different ratios did not influence the modelling results. When compared with the evenly spaced visual system, VS 660 (unevenly spaced) had significantly higher contrast values in all comparison groups (Supplementary Information 4). This indicates that even spacing of the photoreceptors does not improve contrast in our dataset and therefore the results are not biased by uneven spacing of spectral sensitivities in our hypothetical visual systems.

Similar to the results of filter-shifted models, for opsin-shifted models the contrast value increased from VS 580 to at least VS 640 but the differences in contrast value between visual systems were smaller (Fig. S13; full results in Supplementary Information 8).

The contrast values in the corresponding comparisons were qualitatively similar in twilight models, but many contrast values were substantially lower and all below 2 JND (Fig. S10; Supplementary Information 7).

## Discussion

Our results highlight the potential benefit of red to far red sensitivity in resource targeting for jewel beetles and insects more generally. Taken together with intracellular recordings^42^, opsin expression^43^, and electroretinogram data^44^, our behavioural experiments suggest that jewel beetles have visual sensitivity to red wavelengths, possibly extending into the far-red (700–750 nm) in *C. dimidiata*. Vision models using hypothetical insect visual systems indicate that having both short and long wavelength sensitive receptors together enhances the chromatic contrast of beetles with flowers and flowers with leaves, and a long wavelength sensitive (LWS) receptor enhances the chromatic contrast of beetles against leaves. The diversity of spectral profiles among our beetle and flower samples, and the robustness of the results to specific model parameter values (e.g. receptor ratios) suggest that red sensitivity may be important in mate location, mate recognition and/or foraging not only for jewel beetles but also other diurnal insects with an LWS receptor. Additionally, the optimal peak sensitivity of the LWS photoreceptor depends on what is being viewed. When comparing beetle colours with both leaves and flowers, the mean contrast values increase with increasing λ_max_ of the LWS receptor up to 660 nm; whereas when comparing flowers against leaves, the mean contrast values stop increasing beyond 640 nm. Physiological constraints, such as a reduction in absolute sensitivity (filter-shifted) or an increase in thermal noise (opsin-shifted), may limit the upper peak sensitivity of the long wavelength receptor. Our modelling results suggest that a limited increase in visual contrast may also contribute to an upper limit in peak sensitivity for diurnal insects.

Spectral characteristics of vegetation likely explain the visual contrast benefits of an LWS and SWS receptor for flower discrimination and an LWS receptor for leaf discrimination. In chromatic vision, each class of photoreceptor detects the relative difference in reflectance spectra of two objects across the photoreceptor’s absorption span. The reflectance of flowers varies greatly; but due to the characteristics of floral pigments, many have sigmoidal spectra with a sharp rise at shorter wavelengths and plateau at longer wavelengths (Fig. 1b;^62^). By comparison, due to the absorbance profile of chlorophyll, leaves have a characteristic reflectance peak between 500 and 600 nm and a sharp rise in reflectance at ca. 690 nm (the ‘red edge’^63^). The combination of a short and long wavelength receptor picks up differences between sigmoidal flower spectra and either leaves or diverse beetle spectra while an LWS receptor spans wavelengths where leaves differ most strongly from many other natural objects. UV signals are known to play a critical role in flower detection and discrimination in insects, particularly for pollinators^64^. Here, we did not test the contribution of the UV sensitive photoreceptor because almost all trichromatic and tetrachromatic insect visual systems have a UV photoreceptor^9,10^. Therefore, our results do not indicate that the UV receptor is unimportant, but suggest that short and long wavelength sensitive receptors together provide additional benefits for discriminating flowers from leaves and insect colours from vegetation.

Shifting the peak of the long wavelength sensitive receptor improved contrast of beetles against leaves and flowers up to 660 nm, whereas contrast of flowers against leaves stopped increasing beyond 640 nm. Almost all variation in beetle spectra occurs beyond 500 nm and often beyond 600 nm. Increasing λ_max_ of the long wavelength sensitive receptor captures the variation in long wavelengths between beetle and leaves/flowers spectra more effectively. By comparison, the 640 nm long wavelength sensitive receptor provides the greatest contrast between flowers and leaves because it minimizes the relative stimulation by leaf reflectance (not capturing the reflectance at green wavelengths or the increase at ca. 690 nm) but maximises the relative stimulation by flower reflectance (capturing both high flower reflectance and low leaf reflectance before 700 nm). This conclusion is based on the average results in each comparison group, though may differ for specific pairs of spectra.

We employed a receptor noise model of colour discrimination to provide an indication of the effect of variation in visual sensitivities. In reality, however, discrimination is influenced by post-receptor processing, can differ between closely related species and even within species and can be context dependent^65,66^. Therefore, the actual detection threshold requires empirical validation and is likely higher than the theoretical threshold of 1 JND, even under ideal viewing conditions e.g. a threshold of 2.3 for honeybees^67^. Recent evidence also suggests that the relationship between JND value and conspicuousness is non-linear such that high JND values (> 10 JND) may be perceived as equally conspicuous^66,68^. Mean contrast values in daylight generally ranged between 5 and 10 JND suggesting that the differences in contrast values among hypothetical visual systems provide a meaningful indication of differences in discriminability. By contrast, under twilight illumination, mean contrast values were generally < 1 JND and all were < 2 JND, indicating that most colours would be indistinguishable in these conditions.

Vision models with hypothetical opsin-shifted long wavelength sensitive photoreceptors indicate similar patterns but smaller contrast differences between visual systems than the filter-shifted equivalent. Moreover, shifting the peaks of the long wavelength sensitive photoreceptor to longer wavelengths produced a greater increase in contrast value for the filter-shifted models than for the opsin-shifted models. For example, the filter-shifted VS 640 has on average 22% higher contrast value than opsin-shifted VS 640. The lower contrast value for opsin-shifted visual systems is likely due to their much broader sensitivity and greater overlap between photoreceptors. Greater overlap between photoreceptors can decrease colour discrimination^69,70^. Most known insect LWS receptors with a peak beyond 600 nm are due to filtering by coloured pigments. These pigments act as long pass filters and shift photoreceptor sensitivity to longer wavelengths with a narrower span than the underlying opsin absorption spectrum, which also has a beta peak. For example, the perirhabdomal pigments of *Colias erate* butterflies shift the peak sensitivity from around 570 nm to 610–660 nm depending on the pigment type and butterfly sex^11^. The same shifting mechanism has been reported in several other butterflies and is likely the mechanism causing red-shift in a jewel beetle, a bumble bee, and a scarab beetle^14,44,48,52^.

The two mechanisms to shift photoreceptor sensitivity (filter-shift and opsin-shift) may entail different trade-offs. The optimal peak sensitivity of LWS receptors may be limited by dark noise (spontaneous fluctuations in the electrical signals of photoreceptors) because spontaneous pigment activation increases with increasing λ_max_, particularly beyond 620 nm^25,26,29,30^. Dark noise will be greater for opsin-shifted photoreceptors due to their broader absorption span that extends to longer wavelengths. By contrast, the use of filters to shift peak sensitivity reduces absolute sensitivity, thereby reducing the signal to noise ratio^70^. Due to our limited knowledge of the effect of reduced sensitivity on perceived chromatic contrast, we did not consider the reduction in absolute sensitivity from filters in the colour discrimination models. Nevertheless, the cost of noise for both opsin and filtering pigment mechanisms is likely one of the important factors limiting far-red sensitivity in insects.

Another potential fundamental constraint on increasing peak sensitivity of the visual pigment is associated with chromophore types. The chromophore is a carotenoid derivative of vitamin A and one of the two components of a visual pigment. Different chromophore types have varied peak sensitivities and their own unique spectral absorption functions. Each of them can bind to different opsin proteins, leading to diverse spectral tuning. Use of an A2 chromophore has been implicated in shifting sensitivity to longer wavelengths in fish and lizards^23,24^. For example, in the Nile tilapia, using a mix of A1 and A2 chromophores leads to longer λ_max_ of long wavelength sensitive photoreceptors than using A1 alone^71^. However, only A1 and A3 but not A2 chromophore types are used by insects^10,72^, and the absorbance spectra of A1- and A3-visual pigments are similar^73^. Limitations associated with the biomolecular nature of chromophores could restrict the increase in λ_max_ of A1-/A3-visual pigment. Therefore, filter-shifts may be the only way for insects to achieve longer peak sensitivity.

Red sensitivity has been described in numerous insect groups, yet its ecological functions remain unclear. Many insects have been shown to have colour vision that they use in essential visual tasks, such as foraging^74,75^, sexual selection^50,76,77^, and oviposition^12^. Insects with long wavelength sensitive photoreceptors are mainly lepidopterans (butterflies and moths) and coleopterans (beetles), though only a minority have long wavelength sensitive receptors^9,10^. We identified that around 70% of insect species with a long wavelength photoreceptor in Briscoe and Chittka^10^ have prominent colour patterns, and it is possible that long wavelength sensitivity may play a role in detection of conspecifics for these species too. Benefits of having a long wavelength sensitive receptor may extend to other visual behaviours, especially tasks requiring discrimination against prevailing foliage background such as finding prey, host plants, young leaves, and shelter or oviposition sites. For example, female *Lycaena* butterflies use long wavelength sensitivity to locate their host plants for laying eggs^76^. Our results suggest visual advantages of having red sensitivity in visually guided behaviours such as foraging and mate search or recognition. However, there is limited enhancement in perceived contrast beyond the peak of 640 nm for flower-leaf discrimination, indicating that an upper limit of peak sensitivity in terms of visual benefit may apply in some contexts. This supports the view that the optimal long wavelength sensitivity may be related to the essential visual tasks of an animal.

## Supplementary Information

Supplementary Information.

## Data Availability

The dataset and R code are available from Dryad Digital Repository (10.5061/dryad.w6m905qnz). An HTML report showing all the code used for visual modelling and visualisation of results can be viewed online at https://luyiwangtw.github.io/LongWavelengthSensitivity/.
