# Supplementary material for: Insect visual sensitivity to long wavelengths enhances colour contrast of insects against vegetation
Source: Sci Rep. 2022 Jan 19;12:982. doi: 10.1038/s41598-021-04702-w (PMC8770459; doi:10.1038/s41598-021-04702-w)
Supplement: Supplementary file 1 — Supplementary Information. [file 41598_2021_4702_MOESM1_ESM.pdf]

Supplementary materials for: Insect visual sensitivity to long wavelengths enhances colour contrast of insects against vegetation

Lu-Yi Wang[1*], Devi Stuart-Fox[1], Geoff Walker[1], Nicholas W. Roberts[2], Amanda M. Franklin[1]

[1]School of Biosciences, The University of Melbourne, Australia

[2]School of Biological Sciences, University of Bristol, BS8 1TQ, UK

[*]Corresponding author (luyiw@student.unimelb.edu.au; luyiwangtw@gmail.com)

Supplementary Information

1. Behavioural assay in living jewel beetles p 2
2. Details of reflectance measurements p 8
3. Cut-off filters for shifting peak sensitivities p 10
4. Vision models of an evenly spaced tetrachromatic visual system p 12
5. Redistribution of photoreceptor numbers p 15
6. Sensitivity analysis p 17
7. Vision models with civil twilight irradiance p 20
8. Vision models of opsin shifted long wavelength sensitive photoreceptors p 27

Supplementary Tables

Table S2. Species list of spectral data p 33

References p 37

Supplementary Information

1. Behavioural assay in living jewel beetles

Intracellular recordings, opsin expression and electroretinogram data indicate that jewel beetles have a LWS photoreceptor with sensitivity potentially extending into the far red [1-3]. However, their visual sensitivities have not been confirmed with behavioural data. We conducted a behavioural assay to confirm long wavelength sensitivity in living jewel beetles.

Material and methods

We conducted bi-choice behavioural experiments based on the positive phototaxis exhibited by jewel beetles [4]. We used a neutral-grey Y-maze as an arena (10 cm high; two 15 cm wide × 20 cm long arms with a 20 cm wide × 15 cm long base; Fig. S1 *A*) with a light stimulus at the end of one arm and no stimulus at the end of the other. The lit arm was shifted between trials to prevent bias in arm preference. To create the light stimulus, we used a light panel with 6 LED diodes (Marubeni America Corporation; Santa Clara, CA, USA) of the same peak wavelength at the same time to increase the intensity with a piece of diffuse paper placed 1 cm in front. The peak wavelengths of the diodes used in this experiment ranged from 590 to 735 nm (Fig. S2) and were performed in the order of: 590, 680, 700, 720, 735, 645 nm (product number: L600-04, L680-04AU, L700-04AU, L720-04AU, L740-04AU, L645-04). The shorter wavelength in the last trial was to ensure that there was no significant fatigue in beetles after all trials and the responses were reliable. Each beetle was exposed to all light stimuli and

received at least 10-minute break between trials. All trials were conducted in a dark room with an indirect dim light (Fig. S3).

Each trial lasted for 3 minutes and started when the beetle was introduced into the base of the Y-maze. We defined a valid choice as the beetle approaching closer than 5 cm to the diffuse paper at the end of the lit arm. If the beetle made a valid choice before 3 minutes, we stopped the trial and recorded the individual as attracted to that wavelength. Otherwise, the trial ended at 3 minutes and a second trial was performed 10 minutes later with same wavelength to ensure the consistency of its response. If the beetle made a valid choice in the second trial, we then recorded the individual as attracted to that wavelength despite its failure in the first trial. If the beetle still failed to make a valid choice in the second trial, we recorded the individual as not attracted to the wavelength (including in the base zone, dark arm, and in the lit arm but not <5 cm to the end). In both trials, we noted if the beetle moved to within 5 cm of the end of the dark arm to determine the rate of false positives. In total, 28 individuals from 2 species (16 *Castiarina dimidiata* and 12 *C. flavopicta*) completed the 6-wavelength trial series. Every trial was video recorded using a camera with night mode (FDR-AX33; Sony Corporation; Minato, Tokyo, Japan).

We used the beetles' responses (attracted / not attracted) in the behavioural trials to obtain a response curve over 590-735 nm by fitting a logistic regression for the 2 species, separately. In the model, we set the response of the beetles as dependent variable, peak wavelength of the light source as a fixed factor, and individual ID as a random factor to account for repeated trials for each individual.

Results

Behavioural experiments confirmed long wavelength sensitivity in both species of jewel beetle. 96% of jewel beetles were attracted by light at 590 nm and c.a. 87% of them still responded to light at 645 nm (*C. dimidiata*: 100% and 96%; *C. flavopicta*, 93% and 78%; at 590 nm and 645 nm) (Fig. S1 *B*). The percentage of the respondents (i.e. those reached <5 cm to the end the lit arm) decreased steadily after 590 nm in *C. flavopicta* down to 735 nm, whereas the decrease is more abrupt in *C. dimidiata* after 680 nm. At 700 nm, *C. dimidiata* and *C. flavopicta* had a response rate of 56% and 39%, respectively. The 50% response rate was at c.a. 704 nm for *C. dimidiata* and c.a. 685 nm for *C. flavopicta*. Only one *C. dimidiata* individual responded to light at 735 nm. Compared to the dark arm, *C. dimidiata* and *C. flavopicta* beetles reached the ’choice zone’ of the lit arm 19 and 5 times more often, respectively. This indicated that the valid choices of the beetles were not random movement.

Discussion

True colour vision requires discrimination of colours based on their spectral composition, not by their intensity, and is found in many insects including hymenopterans, lepidopterans, and dipterans [5-7]. To determine whether jewel beetles have colour vision and if the LWS receptor is involved, additional behavioural choice experiments need to be conducted. For example, testing the colour vision of jewel beetles by altering the brightness of different peak-wavelength LEDs at two arms respectively in a bi-choice behavioural experiment. The involvement of the LWS receptor could be tested if long-wavelength LEDs are used in one of the above mentioned arms. Furthermore, our experiments were conducted in dimmer lighting

conditions than natural illumination. When eyes are dark adapted, it is possible to see longer wavelengths of light than would be detectable in bright illumination. Additional experiments are required to determine long wavelength sensitivity in natural lighting conditions. However, taken together with the intracellular recordings, opsin expression data and ERG data [1-3], our behavioural experiment suggests that jewel beetles have long wavelength sensitivity.

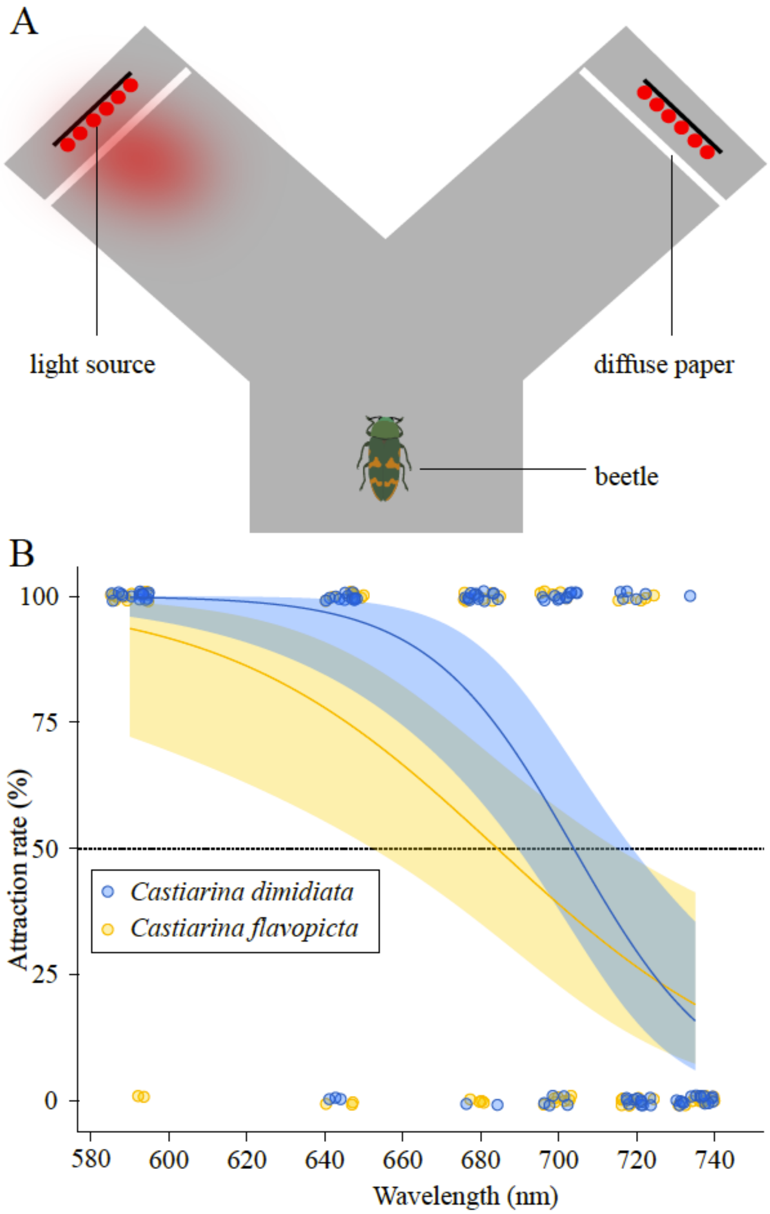

Figure S1. Test of long wavelength sensitivity in jewel beetles. *A*, Y-maze used in the behavioural experiment. *B*, Results of the behavioural experiments on two jewel beetles (*Castiarina dimidiata* and *C. flavopicta*) respectively. Logistic regression lines show beetles' high response rate at 700 nm. 95 % confidence intervals in shaded regions. Figure S1 *A* was created using Inkscape version 1.0.2 (https://inkscape.org); figure 1B was created using R software version 3.6.3 (https://www.r-project.org) and modified using Inkscape.

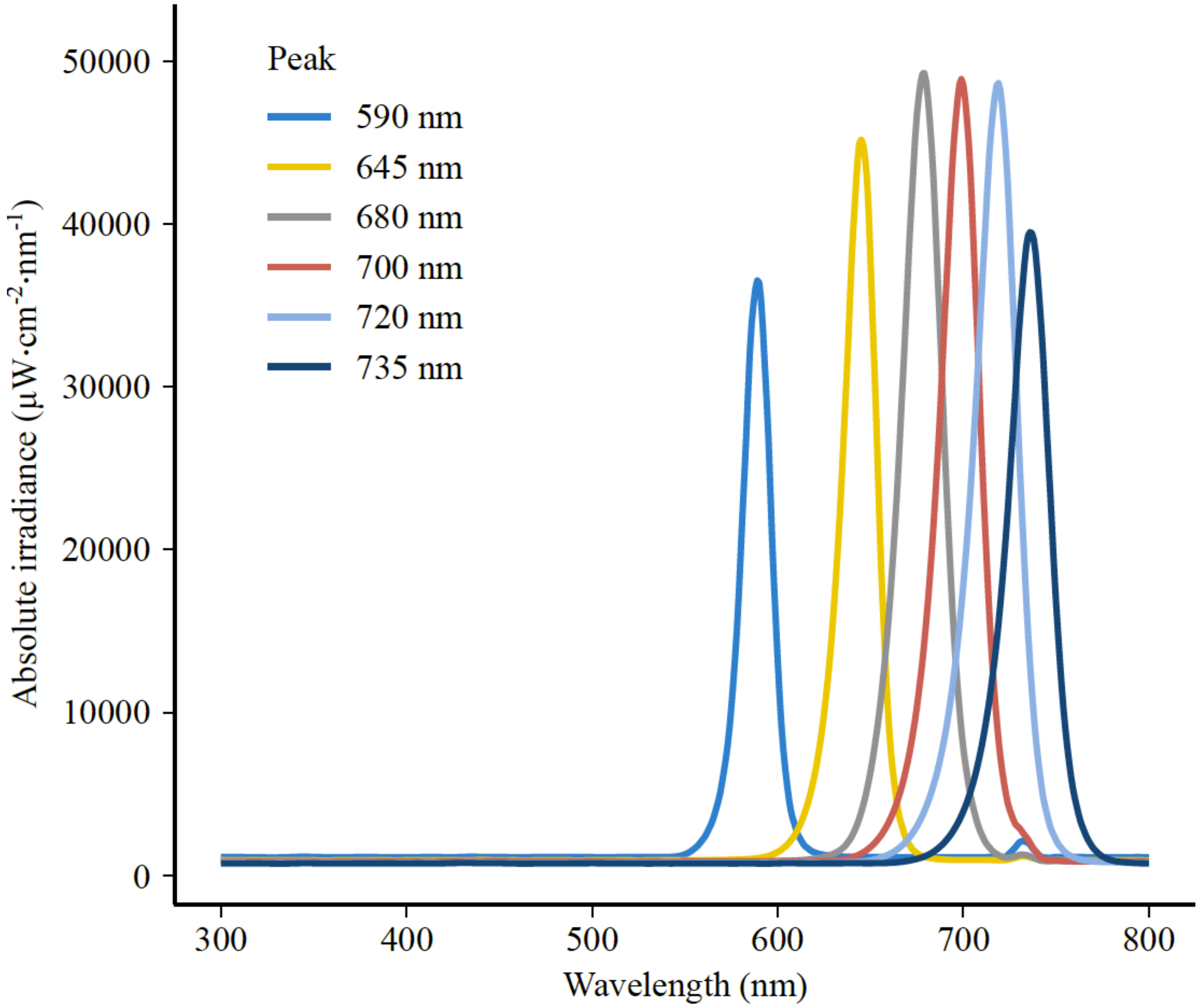

Figure S2. Relative irradiance of the LED lights used in behavioural trials.

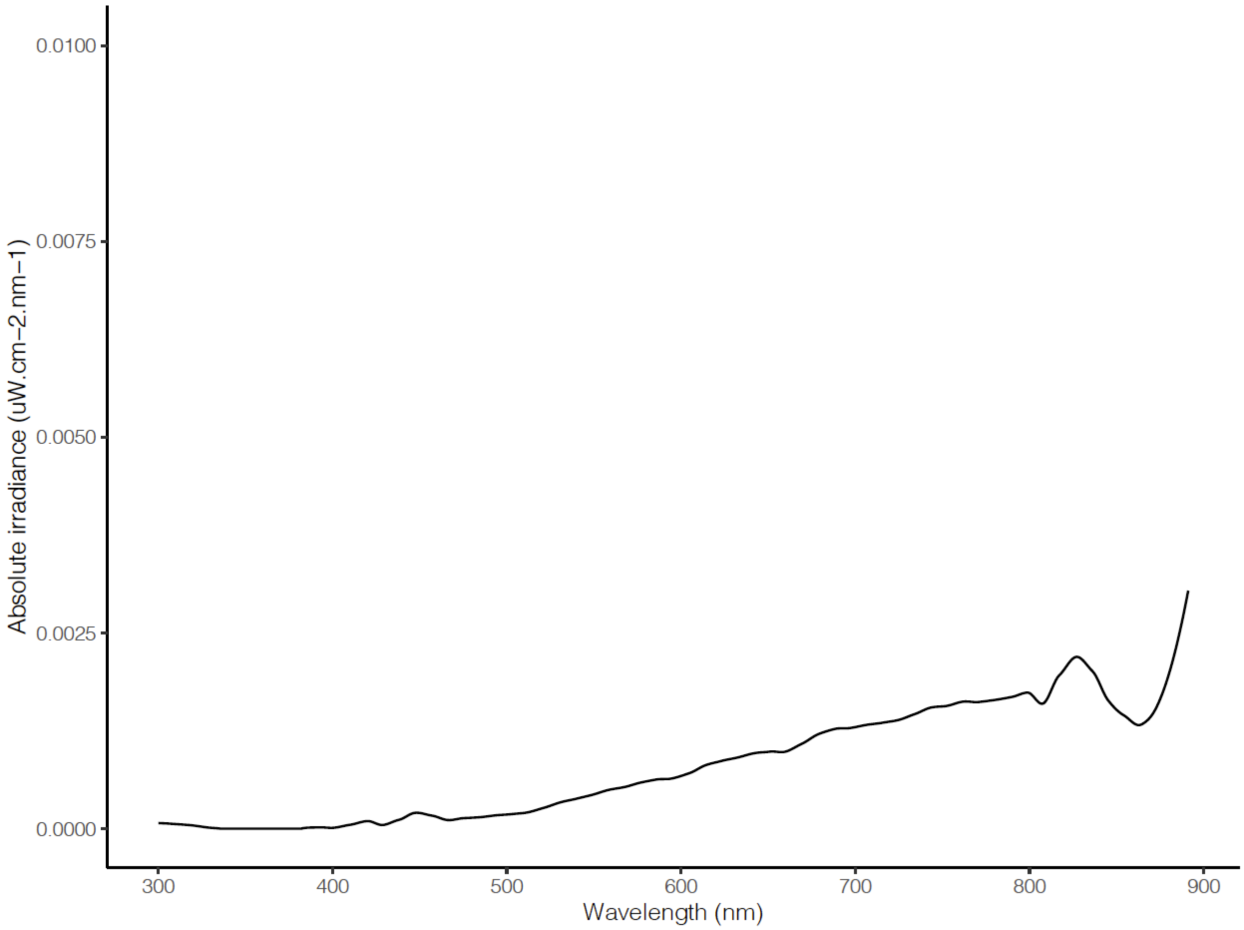

Figure S3. Absolute irradiance of ambient dim lighting in behavioural trials. The light bulb (BS EN 60432-1; Status International Ltd., Cleckheaton, England, UK) was placed inside a wooden box with the top side open and covered with two layers of diffuse paper (Armada Baking paper; Woolworths, Bella Vista, NSW, Australia).

2. Details of reflectance measurements

We measured total reflectance (specular and diffuse; 400-800 nm) using an integrating sphere with an inbuilt tungsten-halogen light source (ISP-REF; Ocean Optics Inc., Dunedin, FL, USA.) and USB2000+ spectrometer (Ocean Optics Inc.) over a 4-mm diameter sampling area. The total specular + diffuse reflectance (i.e. over all angles) measured using an integrating sphere enabled us to capture the average reflectance of the beetles and plants.

Structural colours in beetles can be iridescent (angle-dependent hue shift) or highly specular (angle-dependent intensity shift). However, most beetles used in this study show little or no iridescence and estimates of chromatic contrast from models of colour discrimination are independent of total intensity and therefore unaffected by specularity. Our measurement approach captures chromatic variation in jewel beetle colours, allowing us to model perceived chromatic contrast of these colours against flowers and leaves.

Due to the in-built light source, integrating sphere measurements could not include UV wavelengths. Therefore, to extend the reflectance measurement into the UV range (300-400 nm), we measured reflectance using the same spectrometer with a PX-2 pulsed Xenon light (Ocean Optics Inc.) at 45˚ angle (RPH-1 probe holder; Ocean Optics Inc.; measured area: 5 mm × 3 mm oval). The RPH-1 probe holder is a relatively large, heavy anodized block (with a hole for the probe set at 45˚ relative to the surface) that ensured that the plant measurement surfaces were flat. However, beetle elytra are curved so we had to use a different setup (same spectrometers) to obtain a smaller measuring surface area. This is a custom set-up on an optical bench enabling measurement of 1-mm diameter circle with the source and detector at 30˚

measurement angle. The different measurement angles are unlikely to affect results because the beetles and plants (leaves and flowers) in this study are not iridescent (or only very weakly) and have limited UV reflectance. All measurements were calibrated against a 99% diffuse reflectance white standard (Labsphere, North Sutton, NH, USA). The reflectance spectra were stitched at 400 nm after normalising the magnitude of the reflectance in the vis-NIR range and smoothed using the smoothing function (span = 0.1) in the R package 'pavo' [8].

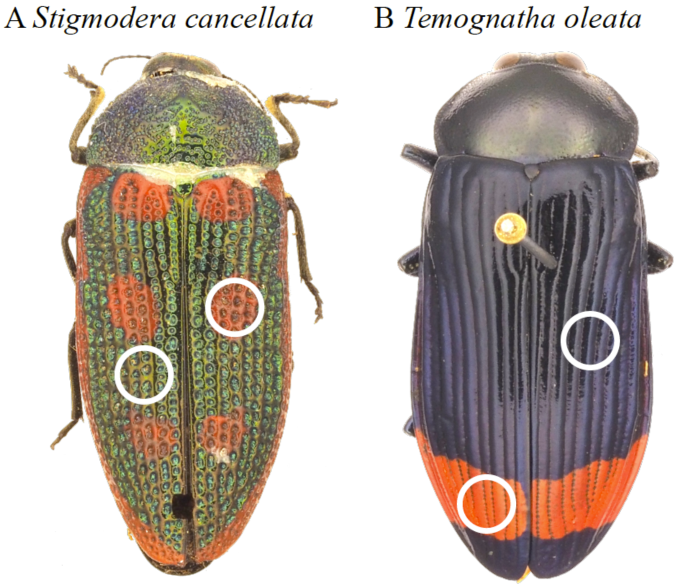

Figure S4. Sampling regions of representative beetles with multiple colours measured. The white circles in the figure are indications of measuring locations. *A*, human-perceived red and green measured for *Stigmodera cancellate*. *B*, human-perceived red and purple measured for *Temognatha oleata*. Photographs by L.-Y.W. and the figure was modified using Inkscape version 1.0.2 (https://inkscape.org).

3. Cut-off filters for shifting peak sensitivities

To obtain the sensitivity curves for LWS photoreceptors, we first generated a 570 nm A1-based profile [9]. Next, we applied different cut-off filters to achieve five peak sensitivities of 580, 600, 620, 640, 660 nm. The cut-off filter was generated from the equation (1) [10]:

$$exp\{-exp[-0.02(\lambda_i - \lambda_0)]\} \tag{1}$$

where λ_i is the wavelength i from 300-800 nm, λ_0 is the wavelength at which the transmittance is equal to $1/e$. We used a λ_0 of 519, 592, 639, 676, 712 to generate sensitivity curves with peak sensitivity of 580 nm, 600 nm, 620 nm, 640 nm, 660 nm. While this filter template was generated for bird oil droplets [10], it was selected because it results in a spectral profile that is similar to the LWS photoreceptor in many insects [3,11-13].

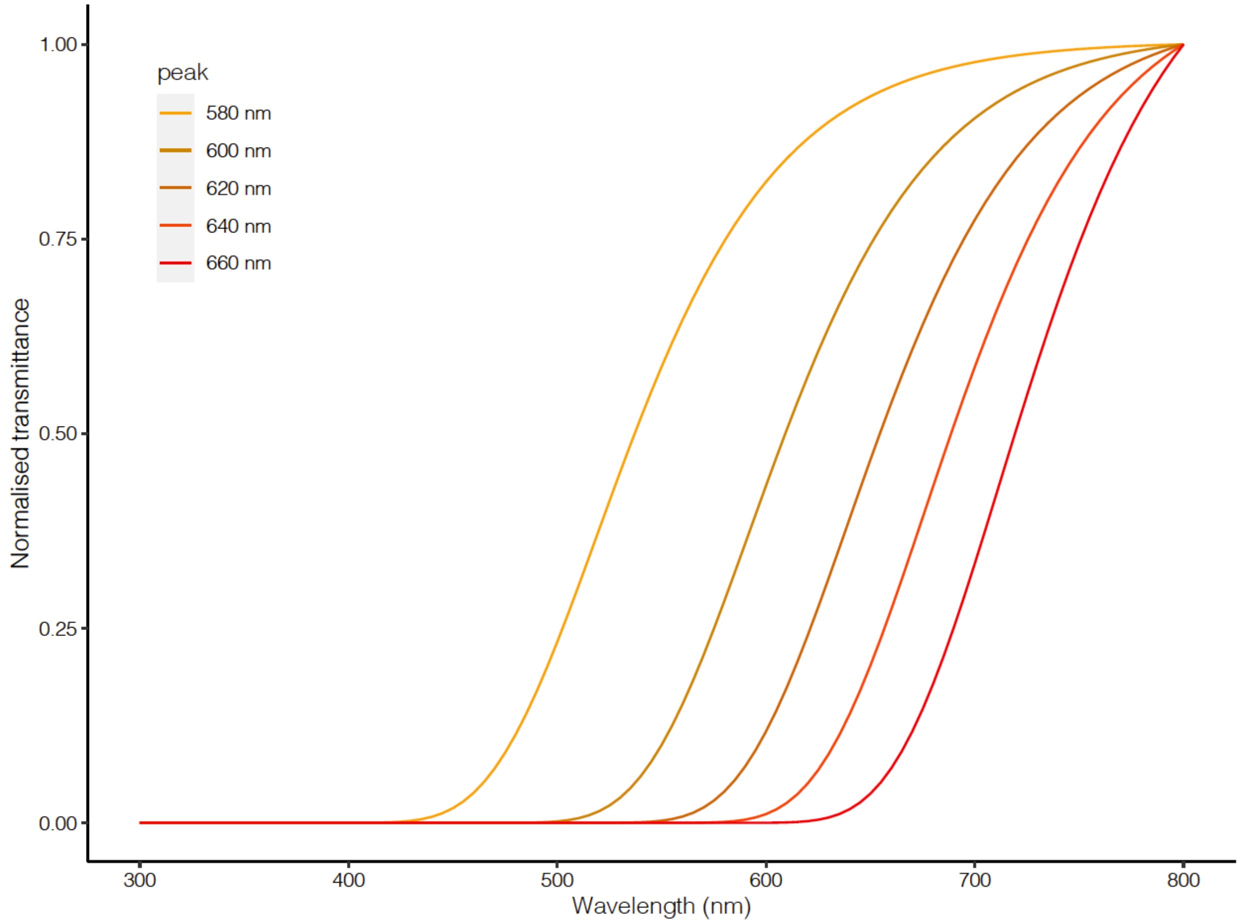

Figure S5. Cut-off filters used for generating filter-shifted LWS photoreceptors. Colours from yellow to red represent filters used to generate LWS photoreceptors with peaks from 580 nm to 660 nm.

4. Vision models of an evenly spaced tetrachromatic visual system

We compared vision models with an evenly spaced visual system and an unevenly spaced visual system, then compared spectral contrast in all three comparison groups (*'flower vs. leaf'*, *'beetle vs. leaf'*, *'beetle vs. flower'*). The evenly spaced visual system has peak sensitivities at 355 nm, 455 nm, 560 nm, and 660 nm (Fig. S6), while VS 660 is used to represent the unevenly spaced visual system, with peak sensitivities at 355 nm, 445 nm, 530 nm, and 660 nm. The model parameters and the statistical methods are the same as described in the main text.

For all three comparison groups, VS 660 (unevenly spaced visual system) has significantly higher contrast than the evenly spaced visual system (Wald Chi-square test, '*flower vs. leaf'* χ^2 = 9.80, *'beetle vs.* leaf' χ^2 = 16.88, *'beetle vs. flower'* χ^2 = 5.05, all $p < 0.001$; table S1). This indicates that the minimal improvement or lack of improvement in contrast beyond 640 nm shown in the main text is not the result of uneven spacing between photoreceptors.

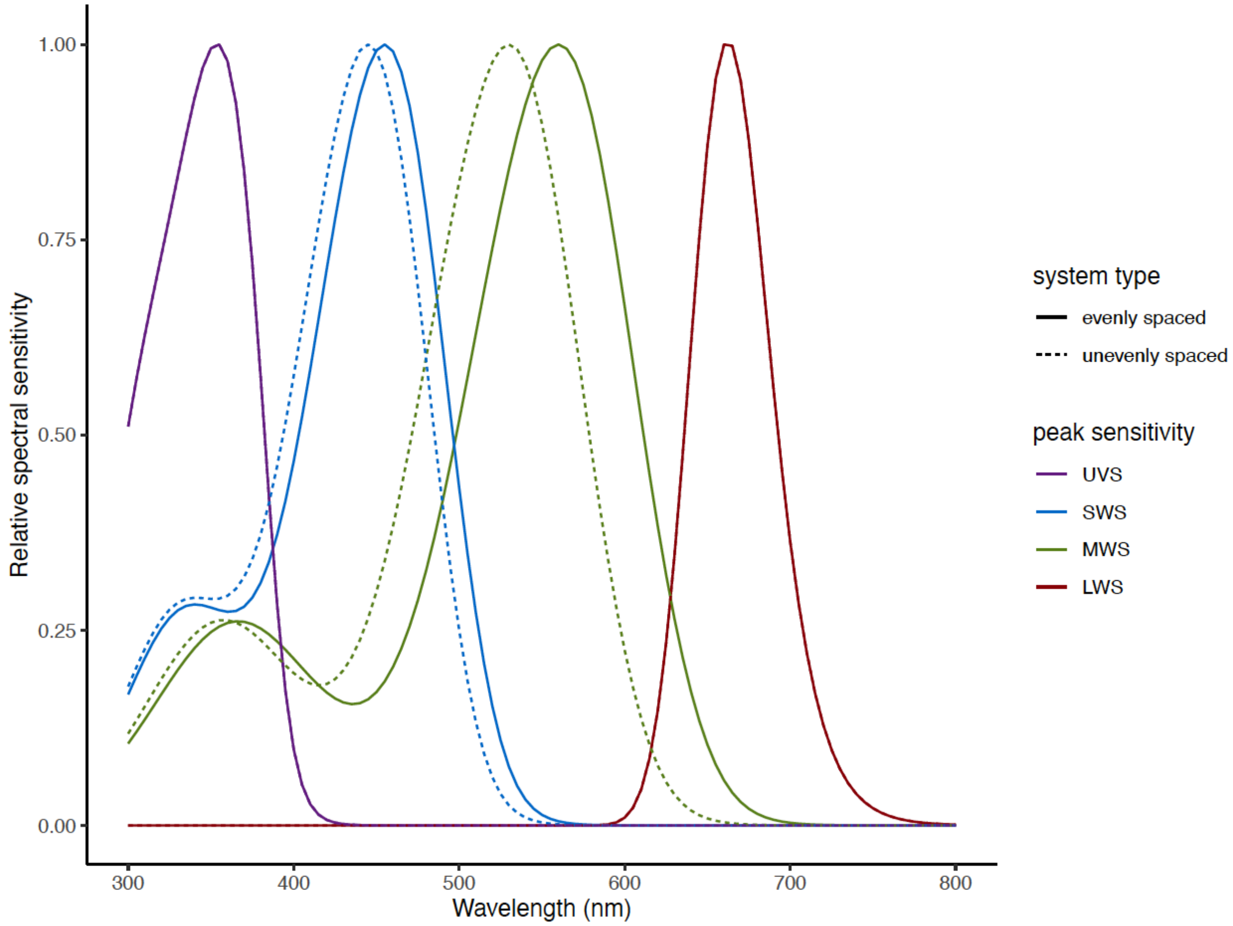

Figure S6. Sensitivity distribution of the evenly and unevenly spaced visual systems. The sensitivity curves are generated using the template from Govardovskii, et al. [9] with a cut-off filter [equation (1) in Supplementary Information 3] applied to create the long wavelength sensitivity curve. The solid curves show the sensitivities used for the evenly spaced visual system and the dotted curves shows those of the unevenly spaced visual system (VS 660).

Table S1. Average contrast (just-noticeable-differences, JND) and the range (minimum – maximum) of the evenly and unevenly spaced tetrachromatic visual system in three comparison groups.

	Flower vs. Leaf	Beetle vs. Leaf	Beetle vs. Flower
Evenly spaced	7.44 (0.52 – 20.94)	6.19 (0.99 – 22.76)	7.68 (0.25 – 21.76)
Unevenly spaced	7.55 (0.50 – 21.78)	6.35 (0.96 – 22.52)	7.82 (0.56 – 21.79)

5. Redistribution of photoreceptor numbers

To compare the performance of trichromats and tetrachromats, we ensured the same number of photoreceptors within a neurally integrative unit. Specifically, we proportionally redistributed photoreceptors from the missing receptor channel between the remaining three channels while keeping the noise level of all photoreceptors the same across visual systems, thereby improving their signal-to-noise ratios [14]. The redistribution of photoreceptor number for trichromats was calculated using equation (2):

$$\eta_i' = (\eta_{lost} \times P_i) + \eta_i \tag{2}$$

where, η_i is the number of cells of type i (i.e. UVS, SWS, MWS, LWS) within a neurally integrative unit, P_i is the proportion of receptor type i among the remaining receptor channels, η_{lost} is the number of cells of the receptor type that was removed, and η_i' is the new number of cells of type i.

Photoreceptor density for the tetrachromat UVS: SWS: MWS: LWS was 1.14: 1: 1.26: 1.38 [2]. These numbers were used for redistribution calculation and the new numbers were 1.6027: 1.4059: 1.7714 for USM, 1.4416 1.5933:1.7451 for UML, and 1.5481: 1.3580: 1.8740 for USL.

After the redistribution of photoreceptors, we adjusted the Weber fraction for each trichromat to ensure that each photoreceptor has the same standard deviation of noise, v_i, as the tetrachromat using equation (3).

$$\omega_i = \frac{v_L}{\sqrt{\eta_i{}'}} \tag{3}$$

where ω_i is the noise for receptor channel i (Weber fraction), and v_L is the standard deviation of the noise in the LWS receptor. We set ω_L to be 0.12 [15] for the LWS receptor in the tetrachromat and derived v_L to be 0.141 using equation (3). This number (v_L = 0.141) was next used to calculate the adjusted Weber fractions for the longest receptor in each trichromat (MWS in USM, LWS in UML and USL) using equation (3) to keep the noise level constant across visual systems.

The new photoreceptor density numbers and the adjusted Weber fractions of the photoreceptor with the longest wavelength in trichromats were then incorporated into the vision models.

6. Sensitivity analysis

To test whether the modelling results were sensitive to the photoreceptor ratios used, we repeated analyses using published ratios that represent some of the known variation in insects. Specifically, we repeated the set of models where tetrachromats have λ_{max} of the LWS photoreceptor increasing from 580 nm to 660 nm. We applied the same modelling method described in the main text but using the known ratios of different tetrachromatic insects, including the jewel beetle, *Agrilus planipennis* (same as the ratio used in the main text; 1.14: 1: 1.26: 1.38 [2]; UVS: SWS: MWS: LWS), and two butterflies, *Papilio xuthus* (1.00: 1.00: 4.08: 2.92 [16]) and *Heliconius sp.* type III (1.00: 1.44: 2.22: 11.11 [17]). The results were qualitatively the same for different photoreceptor ratios among three comparison groups – contrast values increased as the peak sensitivity of LWS photoreceptors increased from 580 to at least 640 nm (20 nm increments). The contrast values continued increasing from VS 640 to VS 660 for the *'beetle vs. leaf'* and *'beetle vs. flower'* but decreased for the *'flower vs. leaf'* (Fig. S7).

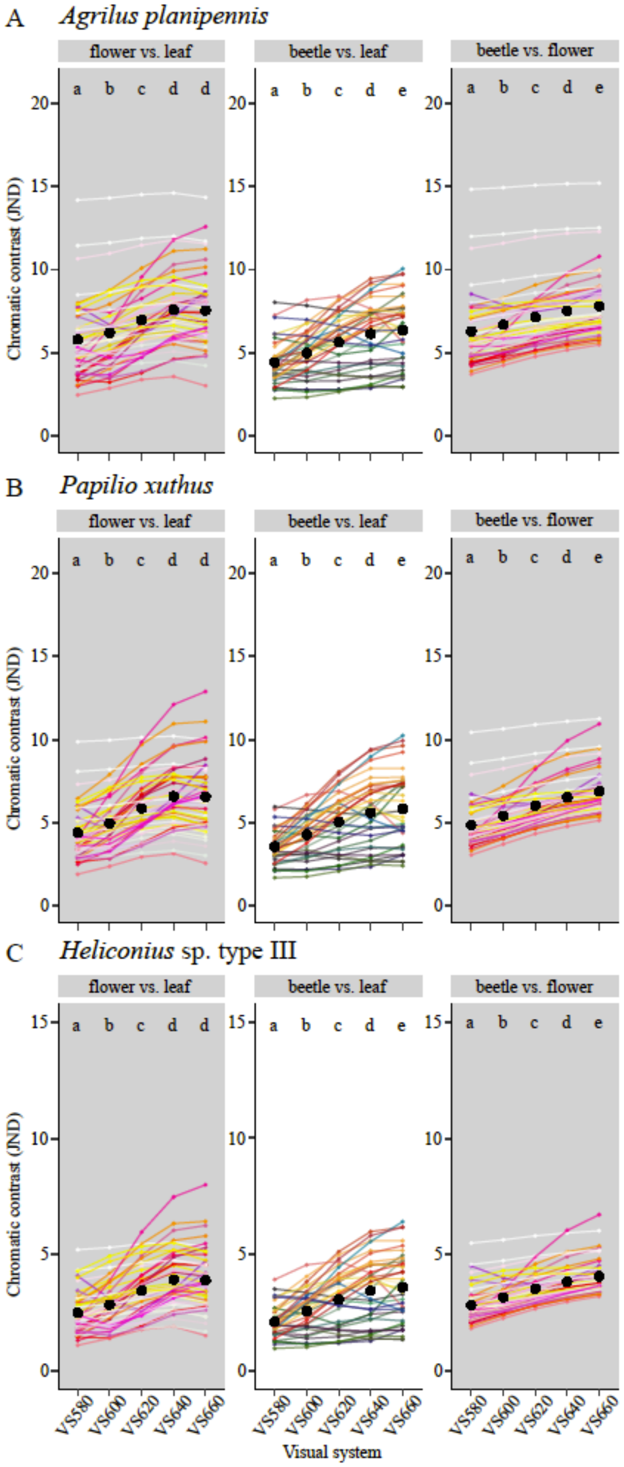

Figure S7. Comparison of the contrast from vision models (VS) using different photoreceptor ratios. *A*, jewel beetle, *Agrilus planipennis*. *B*, butterfly, *Papilio xuthus*. *C*, butterfly, *Heliconius sp.* type III. Black dots show the means of the representative contrast values in visual systems.

Each coloured dot represents the average contrast value of each flower pattern to all leaves (left panel), each beetle colour to all leaves (middle panel) or each flower colour to all beetle colours (right panel). Colours of the dots correspond to the human-visible colour of the flower (left and right panels) or beetle (middle panel) with the lines of the same colour connecting the results between different visual systems. This is for graphical representation only; statistical tests are based on all pairwise combinations of spectra and not averages. Letters on the top of each panel show the significant difference in contrast value between visual systems. Figure S7 *A-C* were created using R software version 3.6.3 (https://www.r-project.org) and modified using Inkscape version 1.0.2 (https://inkscape.org).

7. Vision models with civil twilight irradiance

Changes in illumination can change the contrast between two objects and plays a key role in perception. Here, we tested whether the difference in illumination between daylight and twilight affects contrast. We chose civil twilight because it has the greatest difference in spectral distribution from daylight.

Measurement of twilight illumination

Civil twilight irradiance was measured on a clear day in Alpine National Park, Australia (-37.476930, 146.983360) at 18:15 on September 14th 2019 (Fig. S8). The location was selected to minimize the effect of anthropogenic light. The spectrum was taken using a JAZ spectrometer with inbuilt calibration from the manufacturer and a direct-attached cosine corrector (Ocean Optics Inc). During the measurement, the cosine corrector was held horizontally to collect radiation within 180˚ view from the sky.

Visual modelling

We ran the same vision models and analyses described in the main text but used twilight for illumination instead of daylight. Additionally, we took photon shot noise into account by using ‘quantum’ instead of ‘neural’ for the ‘noise’ argument in ‘coldist’ function in ‘pavo’ [8]. In this function, irradiance absolute intensity is taken into account and the quantum noise is inversely proportional to the quantum catches of the receptors.

Results

I. Visual systems with varied photoreceptor combination

The contrast values obtained from the four visual systems in the models using civil twilight showed different patterns and were substantially lower than the values obtained from the models using daylight (Fig. S9). However, we still found significant differences between visual systems in contrast value of all comparison groups (Wald Chi-square test, *'flower vs. leaf* $\chi^2 = 817$, *'beetle vs. leaf'* $\chi^2 = 2015$, and *'beetle vs. flower'* $\chi^2 = 740.7$, all $p < 0.001$). In twilight illumination, a long wavelength sensitive photoreceptor significantly improved the contrast in all comparison groups. The average contrast values perceived by UML, USL, and USML in all comparison groups were on average 24%, 29%, and 45% higher than USM, respectively.

Tetrachromacy was advantageous in twilight illumination. The tetrachromat (USML) always perceived significantly higher contrasts than the trichromats (USM, UML, USL) (Tukey HSD, USML –USM, USML – UML, USML- USL, in all comparison groups, all $p < 0.001$; Fig. S9). The average contrast values perceived by USML in the three comparison groups were on average 45%, 17%, and 13% higher than USM, UML, and USL, respectively. Among the three trichromats, USL always perceived the highest contrast value (not statistically different to UML in *'beetle vs. leaf'*) while USM always had the lowest for all comparison groups (all pairwise comparisons between trichromats, Tukey HSD, $p < 0.001$, except USL – UML in *'beetle vs. leaf'*, $p = 1.00$). The average contrast values perceived by USL and UML in the three comparison groups were on average 29% and 24% higher than USM.

II. Shifts in the peak of long wavelength sensitivity

The results using civil twilight showed similar patterns but the contrast values were substantially lower (often < 1 JND) than those of daylight models. Contrast values increased as the peak sensitivity of the long wavelength sensitive photoreceptor increased from 580 nm to at least 640 nm for all comparison groups (Wald Chi-square test, *'flower vs. leaf'* χ^2 = 2576, *'beetle vs. leaf'* χ^2 = 2659, *'beetle vs. flower'* χ^2 = 925.6, all $p < 0.001$; Fig. S10). In the *'beetle vs. leaf'* comparison, the contrast value increased from VS 580 to VS 640 but remained the same from 640 nm to 660 nm (stepwise increase in average contrast value by 19%, 16%, and 8% as the long wavelength sensitive peak increased from 580 nm to 640 nm; Tukey HSD, all pairwise comparisons between VS 580 – VS 640, $p < 0.001$, VS 640 – VS 660, $p = 1.00$). Similar pattern was shown in the *'beetle vs. flower'* comparison, where the contrast value increased from VS 580 to VS 660 (stepwise increase in average contrast value by 10%, 10%, and 6% as the long wavelength sensitive peak increased from 580 nm to 640 nm; Tukey HSD, all pairwise comparisons between VS 580 – VS 640, $p < 0.001$, VS 640 – VS 660, $p = 0.15$). The average contrast value of VS 640 was 49% and 29% higher than that of VS 580 for the *'beetle vs. leaf'* and *'beetle vs. flower'* comparisons, respectively. For the *'flower vs. leaf'* comparison, contrast values increased from VS 580 to VS 640 and especially steeply from VS 600 to VS 620 (stepwise increase in average contrast value by 11%, 15%, 8%, as the long wavelength sensitive peak increased from 580 nm to 640 nm; Tukey HSD, all pairwise comparisons between VS 580 – VS 640, $p < 0.001$, except for VS620 – VS 660, $p < 0.05$). After the increase from VS 580 to VS 640, the contrast value decreased significantly from VS 640 to VS 660 by 5% (Tukey HSD, $p < 0.001$).

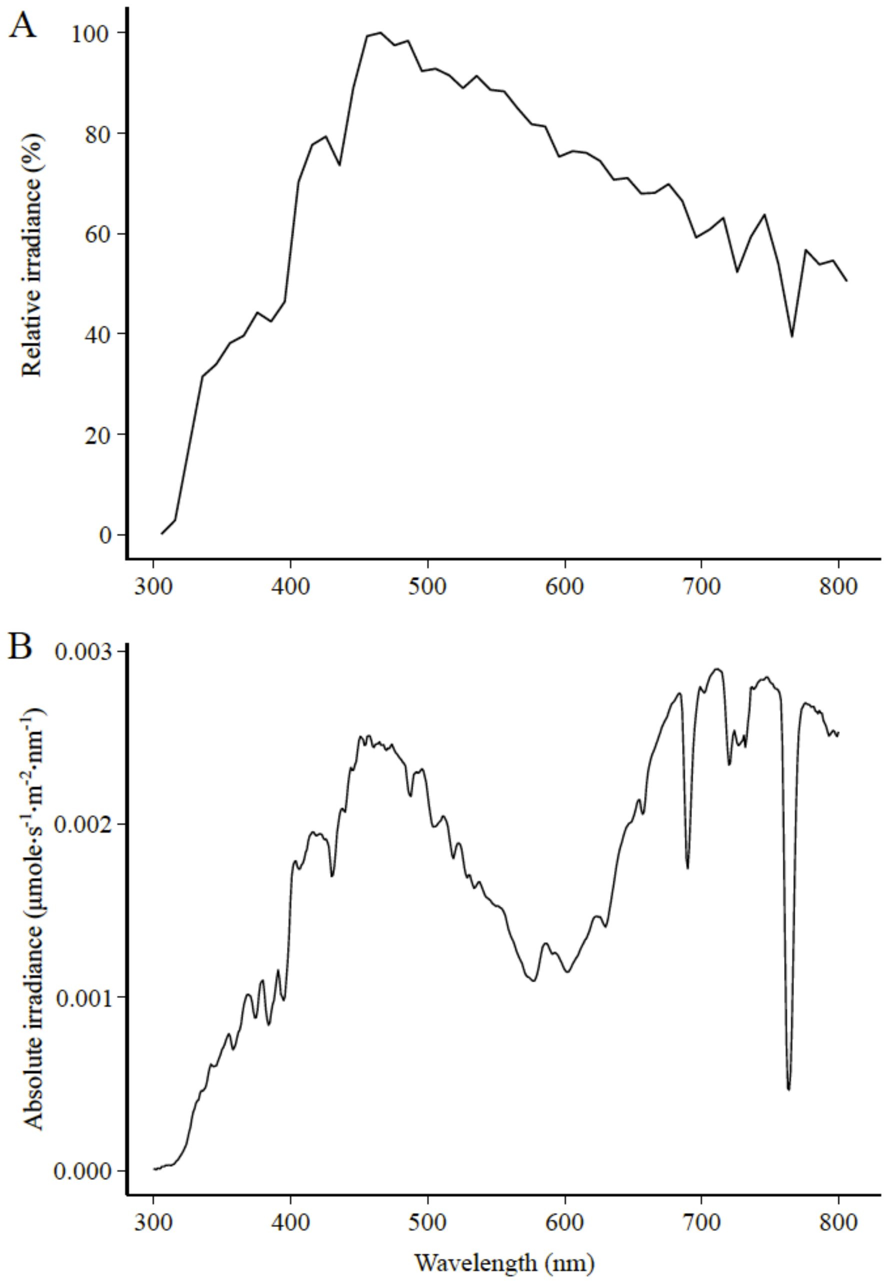

Figure S8. Irradiance of the illuminations used in vision models. A, Relative irradiance of the standard daylight (D65). B, absolute irradiance of civil twilight.

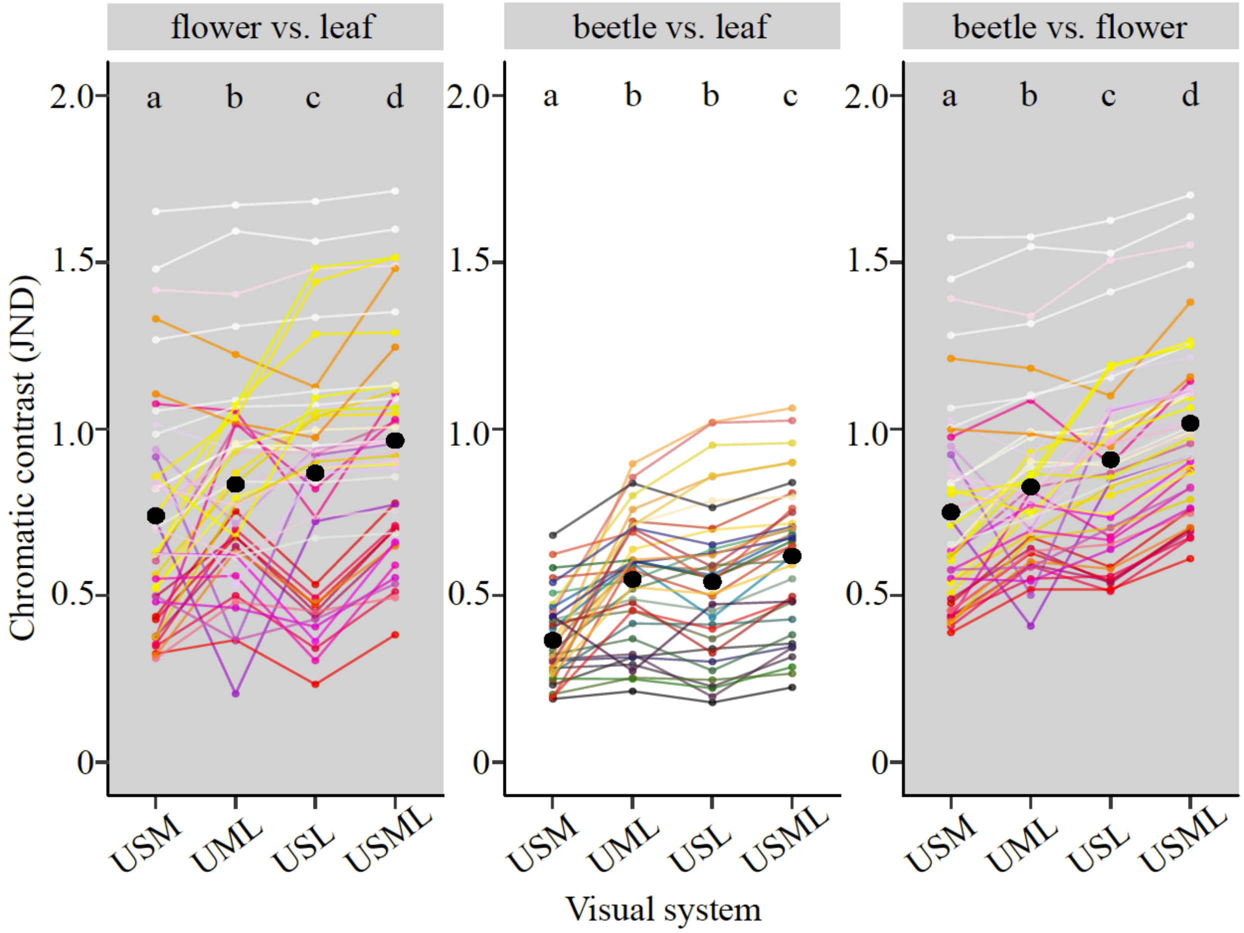

Figure S9. Comparison of chromatic contrast for visual systems with different photoreceptor combinations: USM (UVS, SWS, MWS), UML (UVS, MWS, LWS), USL (UVS, SWS, LWS), USML (UVS, SWS, MWS, LWS) under civil twilight illumination. Black dots show the means of the representative contrast values in visual systems. Each coloured dot represents the average contrast value of each flower pattern to all leaves (left panel), each beetle colour to all leaves (middle panel) or each flower colour to all beetle colours (right panel). Colours of the dots correspond to the human-visible colour of the flower (left and right panels) or beetle (middle panel) with the lines of the same colour connecting the results between different visual systems. This is for graphical representation only; statistical tests are based on all pairwise combinations of spectra and not averages. Letters on the top of each panel show the significant

difference in contrast value between visual systems. The figure was created using R software version 3.6.3 (https://www.r-project.org) and modified using Inkscape version 1.0.2 (https://inkscape.org).

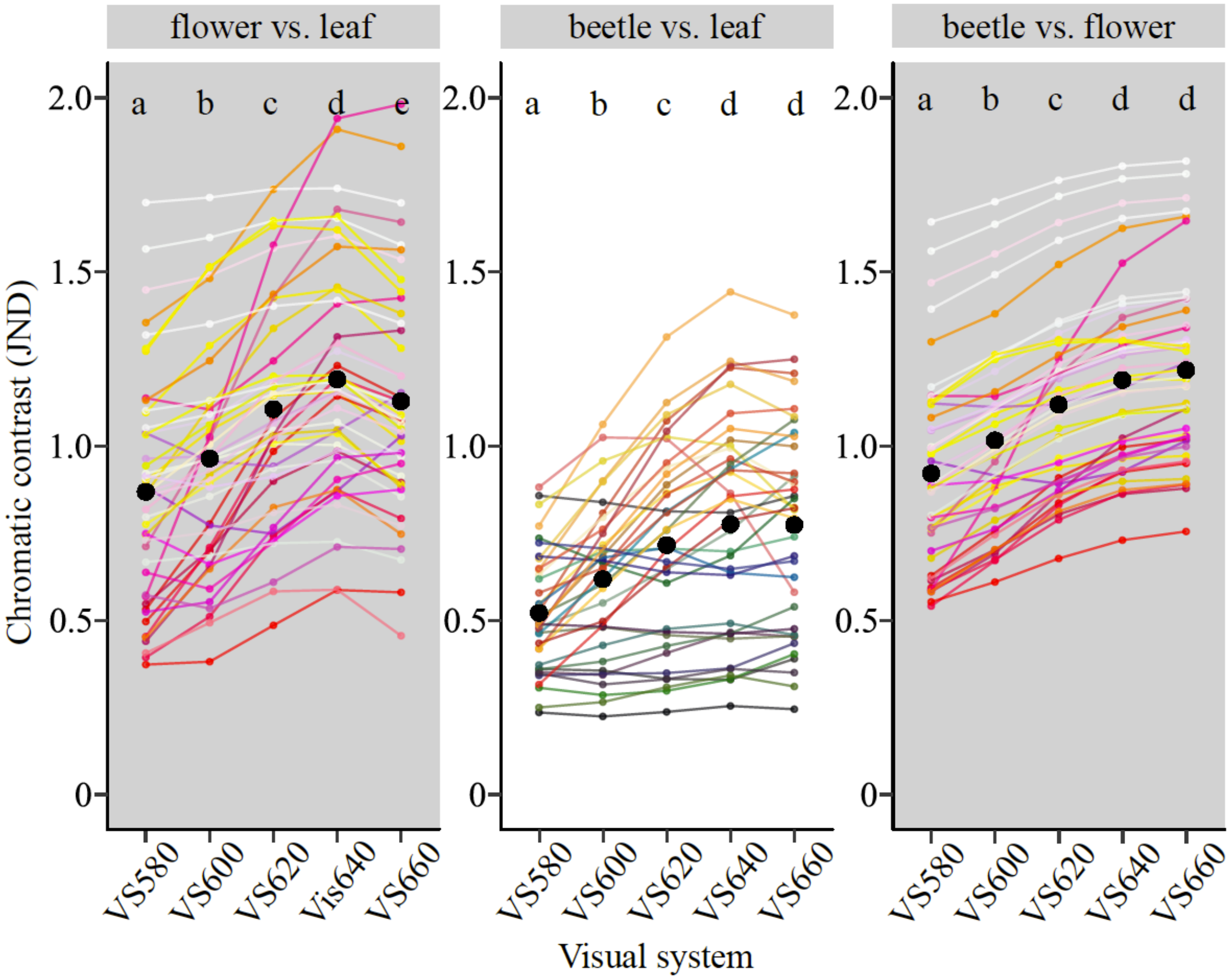

Figure S10. Comparison of chromatic contrast between visual systems (VS) with the long wavelength sensitive photoreceptor peaking at different wavelengths under civil twilight illumination. Black dots show the means of the representative contrast values in visual systems. Each coloured dot represents the average contrast value of each flower pattern to all leaves (left panel), each beetle colour to all leaves (middle panel) or each flower colour to all beetle

colours (right panel). Colours of the dots correspond to the human-visible colour of the flower (left and right panels) or beetle (middle panel) with the lines of the same colour connecting the results between different visual systems. This is for graphical representation only; statistical tests are based on all pairwise combinations of spectra and not averages. Letters on the top of each panel show the significant difference in contrast value between visual systems. The figure was created using R software version 3.6.3 (https://www.r-project.org) and modified using Inkscape version 1.0.2 (https://inkscape.org).

8. Vision models of opsin shifted long wavelength sensitive photoreceptors

Here we created a set of long wavelength sensitive photoreceptors directly from the Govardovskii template [9] and ran the same models described in the main text. Opsin-shifted long wavelength sensitive receptors have much broader sensitivities and beta peaks at shorter wavelengths compared to filter-shifted long wavelength sensitive receptors (Fig. S11). By comparing the modelling results of these two sets of long wavelength sensitive receptors, we show the difference in contrast between the long wavelength sensitive receptor shifts generated from different mechanisms.

Results

I. *Visual systems with varied photoreceptor combination*

We found significant differences between contrast values perceived by different visual systems in of all comparison groups (Wald Chi-square test, '*flower vs. leaf'* $\chi^2 = 46.46$, *'beetle vs. leaf'* $\chi^2 = 147.4$, '*beetle vs. flower'* $\chi^2 = 111.8$, all $p < 0.001$; Fig. S12). The presence of a long wavelength sensitive receptor improved the contrast of beetles against leaves. There were no significant difference between the contrast values of UML, USL, and USML (Tukey HSD, all $p = 1$). All three of these visual systems perceived significantly higher contrast than USM (Tukey HSD, all $p < 0.001$) with the average contrast values all higher than USM by 13%.

The presence of the SWS receptor improved the contrast values of flowers against leaves and beetles against flowers. The average contrast values perceived by the visual systems having a SWS receptor (USM, USL, USML) were 8% higher than the one without (UML) in these

two comparison groups (Tukey HSD, all significant pairwise comparisons, $p < 0.001$; all insignificant comparisons, $p = 1$).

We found no advantage to tetrachromacy. USML perceived insignificantly different contrast value than USM and USL when comparing flowers against leaves and beetles against flowers, and UML and USL when comparing beetles against leaves (Tukey HSD, all $p = 1$).

II. Shifts in the peak of long wavelength sensitivity

Similar to the results of filter-shifted models, contrast values increased as the peak sensitivity of the long wavelength sensitive photoreceptor increased from 580 nm to at least 640 nm for all comparison groups (Wald Chi-square test, *'flower vs. leaf'* $\chi^2 = 181.1$, *'beetle vs. leaf'* $\chi^2 = 634.8$, *'beetle vs. flower'* $\chi^2 = 230.2$, all $p < 0.001$; Fig. S13). However, the difference in contrast value perceived by different visual systems were smaller for opsin-shifted models. In the *'beetle vs. leaf'* comparison, the contrast value increased from VS 580 to VS 660 (stepwise increase in average contrast value by 7%, 7%, 5%, 3% as the long wavelength sensitive peak increased from 580 nm to 660 nm; Tukey HSD, all pairwise comparisons between VS 580 – VS 660, $p < 0.001$, except for VS 640 – VS 660, $p < 0.01$; 13%, 13%, 9%, 3% in filter-shifted models). The average contrast value of VS 640 was 19% higher than that of VS 580 (Tukey HSD, $p < 0.001$). Similarly, in the *'beetle vs. flower'* comparison, the contrast value steadily increased from VS 580 to VS 660 (stepwise increase in average contrast value by 3%, 3%, 4%, 3% as the long wavelength sensitive peak increased from 580 nm to 660 nm; Tukey HSD, all pairwise comparisons between VS 580 – VS 660, $p < 0.01$, except for VS 580 – VS 600, $p = 0.09$; 6%, 7%, 5%, 4% in filter-shifted models), though the contrast value of VS 580 and VS 600 were not

significantly different. For the *'flower vs. leaf'* comparison, contrast value increased steadily from VS 580 to VS 620 and stopped increasing at VS 640 (stepwise increase in average contrast value by 4%, 4%, 1.4%, as the long wavelength sensitive peak increased from 580 nm to 640 nm; Tukey HSD, all pairwise comparisons between VS 580 – VS 640, $p < 0.001$, except VS620-VS660, $p = 0.67$; 7%, 12%, 9% in filter-shifted models) but decreased from VS 640 and VS 660 (Tukey HSD, VS 640 – VS 660, $p < 0.001$, VS 620 – VS 660, $p = 1$).

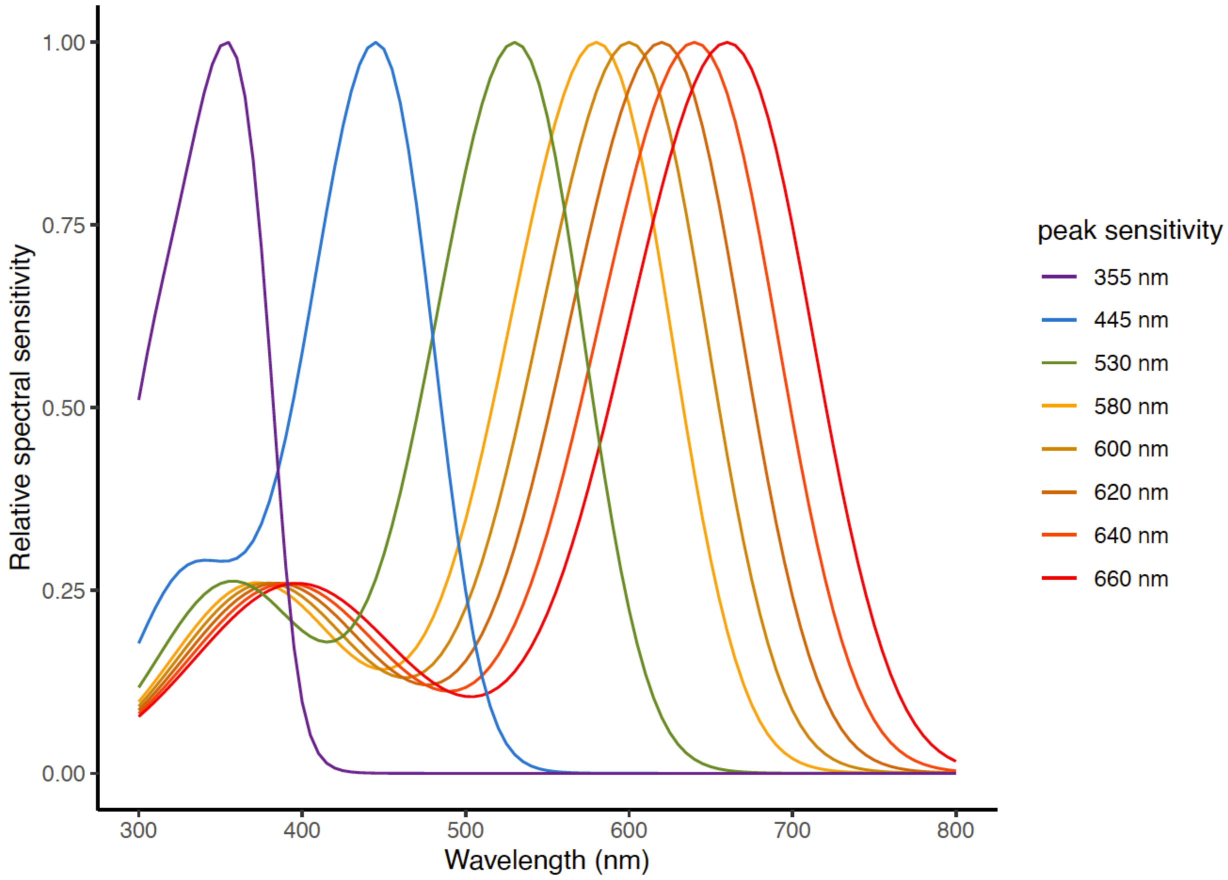

Figure S11. Opsin-shifted sensitivity curves. The long wavelength sensitive photoreceptor sensitivities are generated directly from the Govardovskii template [9] without filters to achieve long wavelength sensitivities from 580-660 nm.

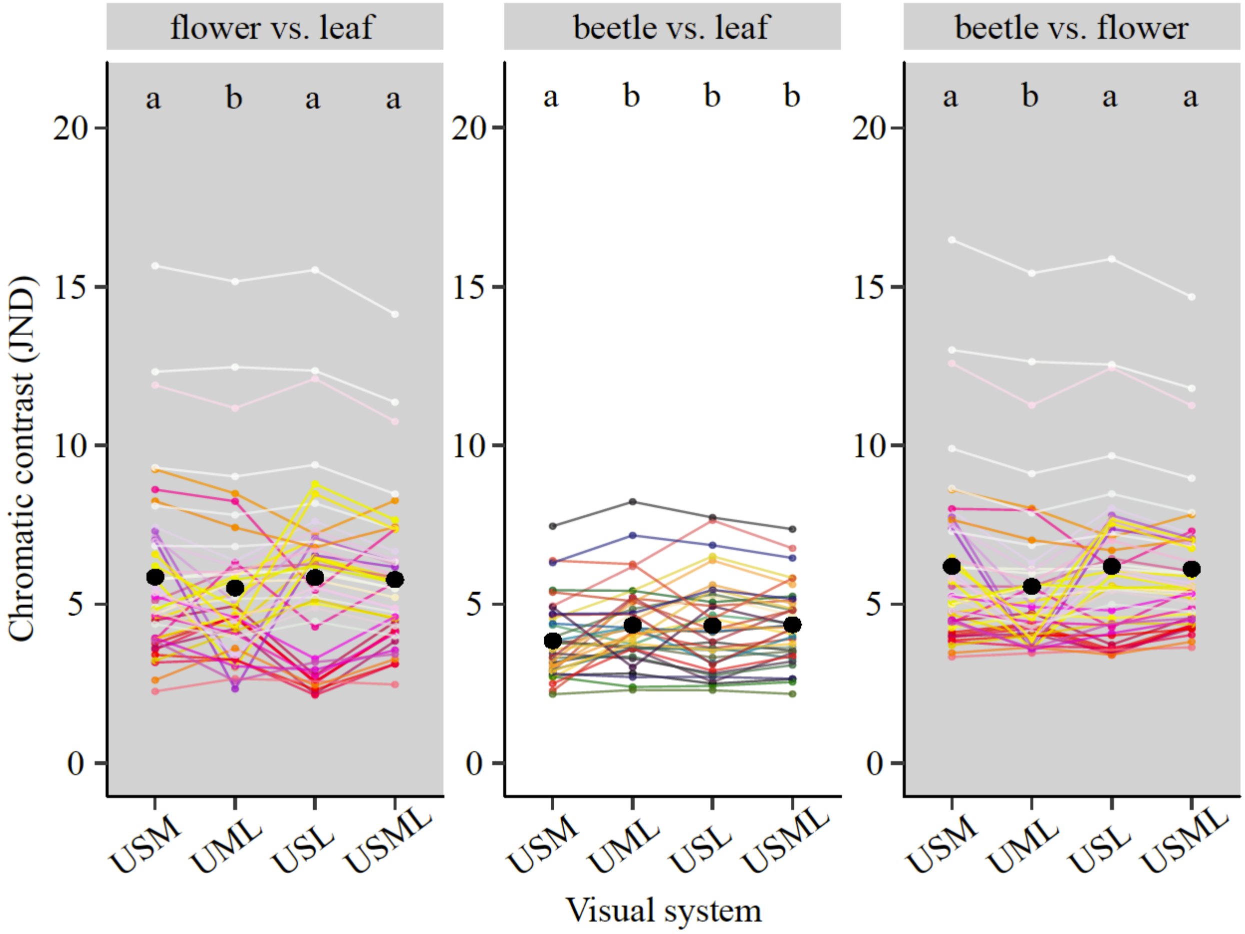

Figure S12. Comparison of chromatic contrast for visual systems with different photoreceptor combinations and the opsin-shifted long wavelength sensitive photoreceptor: USM (UVS, SWS, MWS), UML (UVS, MWS, LWS), USL (UVS, SWS, LWS), USML (UVS, SWS, MWS, LWS). Black dots show the means of the representative contrast values in visual systems. Each coloured dot represents the average contrast value of each flower pattern to all leaves (left panel), each beetle colour to all leaves (middle panel) or each flower colour to all beetle colours (right panel). Colours of the dots correspond to the human-visible colour of the flower (left and right panels) or beetle (middle panel) with the lines of the same colour connecting the results between different visual systems. This is for graphical representation only; statistical tests are

based on all pairwise combinations of spectra and not averages. Letters on the top of each panel show the significant difference in contrast value between visual systems. Three contrasts > 10 JND are from flowers that have high UV - blue chroma compared to beetles and leaves. The figure was created using R software version 3.6.3 (https://www.r-project.org) and modified using Inkscape version 1.0.2 (https://inkscape.org).

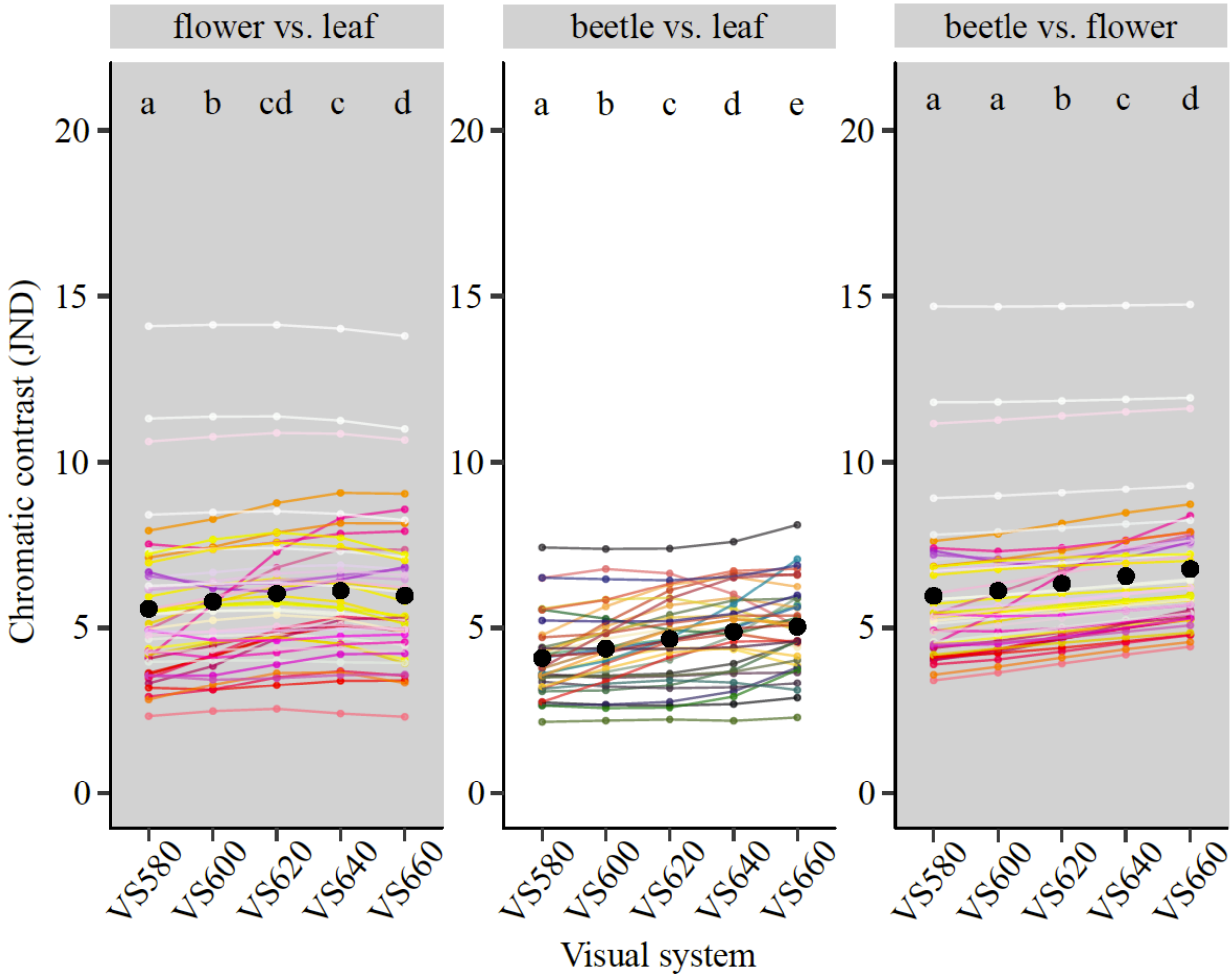

Figure S13. Comparison of chromatic contrast between visual systems (VS) with the opsin-shifted long wavelength sensitive photoreceptor peaking at different wavelengths. Black dots show the means of the representative contrast values in visual systems. Each coloured dot

represents the average contrast value of each flower pattern to all leaves (left panel), each beetle colour to all leaves (middle panel) or each flower colour to all beetle colours (right panel). Colours of the dots correspond to the human-visible colour of the flower (left and right panels) or beetle (middle panel) with the lines of the same colour connecting the results between different visual systems. This is for graphical representation only; statistical tests are based on all pairwise combinations of spectra and not averages. Letters on the top of each panel show the significant difference in contrast value between visual systems. Three contrasts > 10 JND are from flowers that have high UV - blue chroma compared to beetles and leaves. The figure was created using R software version 3.6.3 (https://www.r-project.org) and modified using Inkscape version 1.0.2 (https://inkscape.org).

Supplementary Table

Table S2. Species list of spectral data.

Species	Collection locality	flower/beetle color
Leaves and flowers		
Acacia glaucoptera	-37.691066, 144.589518	yellow
Acacia spilleriana	-37.810256, 145.090334	yellow
Actinotus helianthi	-37.810256, 145.090334	white
Callistemon pallidus	-37.764529, 144.940962	white
Callistemon phoeniceus	-37.691066, 144.589518	red
Calothamnus quadrifidus	-37.691066, 144.589518	red
Chamelaucium 'Lilac Spring'	-37.810256, 145.090334	pink
Chamelaucium uncinatum	-37.691066, 144.589518	pink
Chorizema cordatum	-37.810256, 145.090334	red, orange
Daviesia longifolia	-37.691066, 144.589518	yellow
Epacris impressa 'Bega'	-37.810256, 145.090334	red
Epacris longiflora	-37.810256, 145.090334	red
Eremophila calorhabdos	-37.691066, 144.589518	red
Eremophila georgei	-37.691066, 144.589518	pink
Eremophila hygrophana	-37.691066, 144.589518	purple
Eremophila laanii	-37.691066, 144.589518	white

Eremophila maculata	-37.691066, 144.589518	red
Eremophila nivea	-37.691066, 144.589518	purple
Eriostemon australasius	-37.810256, 145.090334	pink
Eucalyptus celastroides	-37.691066, 144.589518	white
Eucalyptus leucoxylon		
subsp. *leucoxylon*	-37.765049, 144.958947	red
Eucalyptus leucoxylon		
subsp. *pruinosa*	-37.764774, 144.938836	white
Eucalyptus petiolaris	-37.691066, 144.589518	yellow
Eucalyptus sideroxylon	-37.759602, 144.940211	white
Eucalyptus wyolensis	-37.691066, 144.589518	white
Gastrolobium bilobus	-37.691066, 144.589518	orange
Goodenia scapigera	-37.691066, 144.589518	white
Goodenia sp.	-37.691066, 144.589518	yellow
Grevillea asteriscosa	-37.691066, 144.589518	red
Grevillea barklyana	-37.810256, 145.090334	red
Grevillea eriostachys	-37.691066, 144.589518	yellow
Grevillea x *semperflorens*	-37.810256, 145.090334	pink
Leptospermum 'Freya'	-37.810256, 145.090334	pink
Leptospermum liversidgei	-37.810256, 145.090334	white
Melaleuca conothamnoides	-37.691066, 144.589518	pink
Melaleuca fulgens	-37.691066, 144.589518	orange

Melaleuca spathulata	-37.810256, 145.090334	pink
Olearia homolepis	-37.691066, 144.589518	purple
Ozothamnus diosmifolius	-37.810256, 145.090334	white
Phebalium sp.	-37.810256, 145.090334	yellow
Scholtzia capitata	-37.691066, 144.589518	pink
Scholtzia spathulata	-37.691066, 144.589518	pink
Senna artemisioides	-37.810256, 145.090334	yellow
Senna sp.	-37.810256, 145.090334	yellow
Verticordia dichroma	-37.691066, 144.589518	purple
Verticordia plumosa	-37.691066, 144.589518	pink
Beetles		
Calodema regalis	Australian National Insect Collection	yellow
Castiarina australasiae	-37.982090, 145.752880	yellow, black
Castiarina dimidiata	-36.926667, 148.211111	green
Castiarina erythroptera	-37.982090, 145.752880	red
Castiarina ignota	Australian National Insect Collection	red
Castiarina imitator	-36.926667, 148.211111	purple
Castiarina luteipennis	Australian National Insect Collection	yellow
Castiarina punctatosulcata	-37.982090, 145.752880	yellow
Castiarina scalaris	-37.982090, 145.752880	green
Castiarina thomsoni	-37.982090, 145.752880	red, black
Chalcophorotaenia australasiae	Australian National Insect Collection	purple

Cyphogastra farinosa	Australian National Insect Collection	green
Cyria imperialis	Australian National Insect Collection	yellow
Julodimorpha bakewelli	Australian National Insect Collection	yellow
Melobasis cuprifera	Australian National Insect Collection	pink
Melobasis ordinata	-37.559925, 144.298245	green
Melobasis propinqua	-37.884641, 144.181871	green
Melobasis sp	-37.615457, 144.425634	green
Merimna atrata	Australian National Insect Collection	black
Metataenia aurofoveata	Australian National Insect Collection	yellow
Pseudotaenia ajax	Australian National Insect Collection	green
Pseudotaenia gigas	Australian National Insect Collection	green
Selagis olivacea	Australian National Insect Collection	purple
Selagis viridicyanea	Australian National Insect Collection	purple
Stigmodera cancellata	Australian National Insect Collection	red, green
Stigmodera gratiosa	Australian National Insect Collection	blue
Stigmodera macularia	-37.982090, 145.752880	yellow
Temognatha bonvouloirii	Australian National Insect Collection	red, purple
Temognatha bruckii	Australian National Insect Collection	yellow
Temognatha chalcodera	Australian National Insect Collection	red
Temognatha obscuripennis	Australian National Insect Collection	purple
Temognatha oleata	Australian National Insect Collection	red, purple

References

1 Crook, D. J. *et al.* Laboratory and field response of the emerald ash borer (Coleoptera: Buprestidae), to selected regions of the electromagnetic spectrum. *Journal of Economic Entomology* **102**, 2160-2169 (2009).

2 Lord, N. P. *et al.* A cure for the blues: opsin duplication and subfunctionalization for short-wavelength sensitivity in jewel beetles (Coleoptera: Buprestidae). *BMC evolutionary biology* **16**, 107 (2016).

3 Meglič, A., Ilić, M., Quero, C., Arikawa, K. & Belušič, G. Two chiral types of randomly rotated ommatidia are distributed across the retina of the flathead oak borer *Coraebus undatus* (Coleoptera: Buprestidae). *The Journal of Experimental Biology* **223**, jeb225920, doi:10.1242/jeb.225920 (2020).

4 Chen, Y. G. & Poland, T. M. Biotic and abiotic factors affect green ash volatile production and emerald Ash borer adult feeding preference. *Environmental Entomology* **38**, 1756-1764, doi:10.1603/022.038.0629 (2009).

5 Briscoe, A. D. & Chittka, L. The evolution of color vision in insects. *Annual Review of Entomology* **46**, 471-510, doi:10.1146/annurev.ento.46.1.471 (2001).

6 Goldsmith, T. H. Optimization, constraint, and history in the evolution of eyes. *Quarterly Review of Biology* **65**, 281-322, doi:10.1086/416840 (1990).

7 Menzel, R. in *Comparative physiology and evolution of vision in invertebrates: A: invertebrate photoreceptors* (ed H. Autrum) 503-580 (Springer Berlin Heidelberg, 1979).

8 Maia, R., Gruson, H., Endler, J. A. & White, T. E. pavo 2.0: new tools for the spectral and spatial. *bioRxiv* (2018).

9 Govardovskii, V. I., Fyhrquist, N., Reuter, T., Kuzmin, D. G. & Donner, K. In search of the visual pigment template. *Visual Neuroscience* **17**, 509-528, doi:10.1017/s0952523800174036 (2000).

10 Hart, N. S. & Vorobyev, M. Modelling oil droplet absorption spectra and spectral sensitivities of bird cone photoreceptors. *Journal of Comparative Physiology a-Neuroethology Sensory Neural and Behavioral Physiology* **191**, 381-392, doi:10.1007/s00359-004-0595-3 (2005).

11 Martínez-Harms, J. *et al.* Evidence of red sensitive photoreceptors in *Pygopleurus israelitu*s (Glaphyridae: Coleoptera) and its implications for beetle pollination in the southeast Mediterranean. *Journal of Comparative Physiology a-Neuroethology Sensory Neural and Behavioral Physiology* **198**, 451-463, doi:10.1007/s00359-012-0722-5 (2012).

12 Ogawa, Y., Kinoshita, M., Stavenga, D. G. & Arikawa, K. Sex-specific retinal pigmentation results in sexually dimorphic long-wavelength-sensitive photoreceptors in the eastern pale clouded yellow butterfly, *Colias erate*. *Journal of Experimental Biology* **216**, 1916-1923, doi:10.1242/jeb.083485 (2013).

13 Stavenga, D. G. & Arikawa, K. Photoreceptor spectral sensitivities of the Small White butterfly *Pieris rapae crucivora* interpreted with optical modeling. *Journal of*

Comparative Physiology a-Neuroethology Sensory Neural and Behavioral Physiology **197**, 373-385, doi:10.1007/s00359-010-0622-5 (2011).
14 Vorobyev, M., Osorio, D., Bennett, A. T. D., Marshall, N. J. & Cuthill, I. C. Tetrachromacy, oil droplets and bird plumage colours. *Journal of Comparative Physiology a-Neuroethology Sensory Neural and Behavioral Physiology* **183**, 621-633, doi:10.1007/s003590050286 (1998).
15 Vorobyev, M., Brandt, R., Peitsch, D., Laughlin, S. B. & Menzel, R. Colour thresholds and receptor noise: behaviour and physiology compared. *Vision Research* **41**, 639-653, doi:10.1016/s0042-6989(00)00288-1 (2001).
16 Matsushita, A., Awata, H., Wakakuwa, M., Takemura, S. Y. & Arikawa, K. Rhabdom evolution in butterflies: insights from the uniquely tiered and heterogeneous ommatidia of the Glacial Apollo butterfly, *Parnassius glacialis*. *Proceedings of the Royal Society B-Biological Sciences* **279**, 3482-3490, doi:10.1098/rspb.2012.0475 (2012).
17 McCulloch, K. J. *et al.* Sexual dimorphism and retinal mosaic diversification following the evolution of a violet receptor in butterflies. *Molecular Biology and Evolution* **34**, 2271-2284, doi:10.1093/molbev/msx163 (2017).
